# Gender Differences in Posttraumatic Stress Symptoms after a Terrorist Attack: A Network Approach

**DOI:** 10.3389/fpsyg.2017.02091

**Published:** 2017-12-01

**Authors:** Marianne S. Birkeland, Ines Blix, Øivind Solberg, Trond Heir

**Affiliations:** ^1^Norwegian Centre for Violence and Traumatic Stress Studies, Oslo, Norway; ^2^Faculty of Medicine, Institute of Clinical Medicine, University of Oslo, Oslo, Norway

**Keywords:** PTSD, sex differences, gender differences, traumatology, terrorism, network analysis

## Abstract

**Background:** Posttraumatic stress symptoms are more prevalent in women than in men. To improve our understanding of gender differences in PTSD, detailed knowledge about the underlying symptom networks and gender specific symptom profiles is needed.

**Objective:** We aimed to describe the gender differences in levels of individual posttraumatic stress symptoms after a terrorist attack, as well as identify possible gender differences in associations between posttraumatic stress symptoms.

**Method:** This study used survey data from ministerial employees directly (*n* = 190) and indirectly (*n* = 1,615) exposed to the 2011 Oslo bombing. Data was collected approximately 10 months after the event. In order to investigate gender differences in levels of symptoms, we used bootstrapped means and standard deviations. Network analyses were conducted to identify gender differences in the associations between posttraumatic stress symptoms.

**Results:** Women reported higher levels of all symptoms, and the strongest effect sizes were found for symptoms of re-experiencing, and anxious and dysphoric arousal. Among individuals with considerable levels of posttraumatic stress symptoms, women reported higher levels of physiological cue activity and exaggerated startle response. No significant gender differences in the networks of posttraumatic stress were found.

**Conclusions:** The present results find no indication that the gender difference in prevalence of PTSD can be explained by differences in associations between symptoms. In order to determine if this finding can be applied to other participants and circumstances, future studies should seek to replicate this study in both community and clinical samples.

## Introduction

After experiencing a traumatic event, women have twice the probability of developing posttraumatic stress disorder (PTSD), compared to men (Kilpatrick et al., 2013; Pineles et al., 2017). This difference cannot be fully explained by different patterns of trauma exposure, such as women experiencing more interpersonal violence such as sexual assaults (Tolin and Foa, 2006). For example, women report higher levels of posttraumatic stress symptoms than men after earthquakes (Carmassi and Dell'Osso, 2016), motor vehicle accidents (Fullerton et al., 2001), and terrorism (Solomon et al., 2005; Sever et al., 2008). Furthermore, studies of measurement invariance of PTSD scales suggest no or minimal gender differences in factor structure of PTSD, meaning that the instruments measure the PTSD symptoms in both genders equally well (Wang et al., 2013; Carragher et al., 2016; Frankfurt et al., 2016).

To understand the gender differences in PTSD prevalence, we need to examine how PTSD is developed and maintained. A core symptom of PTSD is intrusive recollections of the traumatic event (McNally, 2005) and cognitive theories on PTSD have focused on the role of memory in symptom etiology and maintenance (Ehlers and Clark, 2000; Rubin et al., 2008). Cross-sectional studies examining networks of posttraumatic stress symptoms have found that re-experiencing symptoms, especially physiological reactivity, were central to other symptoms (Armour et al., 2017; Bryant et al., 2017; McNally et al., 2017). Moreover, several of these studies found a strong association between physiological reactivity and hyperarousal symptoms (Bryant et al., 2017; McNally et al., 2017), and this association was found to be stronger 12 months after trauma, than in the acute phase (Bryant et al., 2017). In another study, re-experiencing symptoms such as recurrent or distressing recollections and dreams in the acute phase were predictive of PTSD diagnosis 6 months after the traumatic event (Haag et al., 2017). Thus, the current cross-sectional network studies indicate that becoming physiologically reactive and upset in response to reminders of the trauma may be key drivers of other symptoms. In accordance with this, longitudinal studies using cross-lagged panel designs have also indicated that re-experiencing and hyperarousal symptoms preceded the other symptom clusters of PTSD (Schell et al., 2004; Marshall et al., 2006; Solomon et al., 2009; Pietrzak et al., 2014; Solberg et al., 2016). Thus, one possibility is that gender differences in development of posttraumatic stress may be, at least partially, attributed to differences in how men and women remember or re-experience the traumatic event.

Only a few studies have explored gender differences in individual symptoms or clusters of symptoms. Fullerton et al. (2001) showed that 1 month after a serious motor vehicle accident, women reported a higher level of re-experiencing symptoms when facing situations similar to the accident, and a higher level of physical reactivity when remembering the accident. In a study focusing on the relationships between sleep and posttraumatic stress symptoms after being injured, women reported more nightmares and sleep-interfering disruptive nocturnal behaviors, especially hot flashes and memories/nightmares of trauma, compared to men (Kobayashi and Delahanty, 2013). Furthermore, in a study of the general population living in New York City after September 11, 2001, women reported significantly more re-experiencing and hyperarousal symptoms than men (Stuber et al., 2006). No differences were found in avoidance/numbing symptoms. Taken together, these studies suggest that especially re-experiencing symptoms seem to be more pronounced in women.

Gender differences in fear processing may contribute to the higher level of arousal symptoms as well as the higher PTSD prevalence in women. Experimental studies have found that women show more reactivity than men in neural networks associated with fear and arousal responses (Felmingham et al., 2010), and greater differential conditioned skin conductance responses to aversive stimuli (Inslicht et al., 2013). It has been proposed that hyperactivation of neural fear processing networks can explain gender differences in PTSD prevalence (Olff et al., 2007). Furthermore, women also have higher risk perceptions (Lerner et al., 2003; Kung and Chen, 2012), more catastrophic cognitions, and higher sensitivity to anxiety (fear of anxiety-related sensations) than men (Armstrong and Khawaja, 2002). Accordingly, one may argue that symptoms within the re-experiencing cluster, e.g., physiological cue activity, can be more strongly connected to symptoms within the anxious arousal cluster, e.g. feeling easily startled, in women than in men. However, no current study has investigated if the PTSD symptoms interact with each other in different ways among women than men.

In the present study, we therefore wanted to investigate the associations between PTSD symptoms in a sample of men and women directly and indirectly exposed to the 2011 Oslo bombing. In previous studies, we have reported that both directly and indirectly exposed individuals report symptoms of posttraumatic stress 10 months after the terrorist attack (Hansen et al., 2013), and we have described development of the symptoms in these groups over time (Solberg et al., 2016; Hansen et al., 2017). We have also explored the network of posttraumatic stress symptoms in the directly exposed sample (Birkeland and Heir, 2017). In the present study, our aim was twofold; (1) investigate how men and women differed in levels of the 17 individual DSM-IV posttraumatic stress symptoms 10 months after the terrorist attack, among both the directly and indirectly exposed individuals, and (2) identify possible gender-specific differences in the associations between the symptoms. For the last aim, we excluded individuals with very low levels of posttraumatic stress symptoms. We hypothesized that the gender differences would be especially evident for the symptoms that tap into re-experiencing/intrusions symptoms, and that symptoms of re-experiencing would be more strongly connected to anxious arousal symptoms among women.

## Materials and methods

### Participants

The participants were ministerial employees exposed to the 2011 Oslo bombing. On July 22, 2011, a car bomb exploded in the executive governmental quarter. The blast damaged governmental buildings, killed eight people, and injured 209 people. The data for this study were collected 10 months after the attack, in April/May 2012. All of the employees (*N* = 3,520) in 14 of the 17 Norwegian ministries were invited to participate in a research project titled “Mental health and work environment factors in the aftermath of the Oslo terrorist attack July 22nd, 2011” (Hansen et al., 2017). When the bomb went off, 342 people were present at the site of the explosion. Their lives were in danger, and they were thus considered as directly exposed, fulfilling the criterion A for PTSD according to DSM-IV and DSM-5. Due to summer vacation or work assignments elsewhere, 3,178 ministry employees were not present at the site of the bomb explosion. Still, they were part of a work community that was the target of terrorism; they lost colleagues, they were confronted with broken buildings and offices, and they were thus considered as indirectly exposed. Of the directly exposed employees, 207 responded to the survey (60.5%). Among these, 18 individuals did not complete the measure of posttraumatic stress and were excluded. Therefore, the number of participants directly exposed to the traumatic event was 190. Of these, 117 were women and 73 were men. Of the indirectly exposed employees, 1,763 responded to the survey (55.5%). Among these, 148 individuals did not provide complete data on posttraumatic stress, resulting in a sample of 1,615 indirectly exposed individuals, 918 women and 697 men. All of the participants were informed about the purpose and content of the study, and they were given the opportunity to withdraw. The study was approved by the Regional Ethics Committee in Norway.

### Measures

Posttraumatic stress was assessed with the Norwegian version of the posttraumatic checklist, civilian version (PCL-S) (Weathers and Ford, 1996; Hem et al., 2012). The PCL-S is a 17-item self-administered questionnaire that assesses PTSD symptom severity according to DSM-IV. In this version the symptoms endorsed are specifically linked to a traumatic event, and instructions to consider the Oslo bombing of 22 July 2011 when answering were given. The respondents were asked to rate the extent to which they had been bothered by PTSD symptoms over the last 4 weeks on a five-point scale that ranged from “Not at all” (1) to “Extremely” (5). The symptom ratings were summed to reflect a total score. The cutoff for “caseness” for this civilian sample was a sum score ≥30 (U. S. Department of Veteran Affairs, 2012). Cronbach's alpha for the total PCL-S was 0.94.

Confirmatory factor analyses indicated that in line with other studies (Elhai and Palmieri, 2011; Armour et al., 2016), the data fit the five factor dysphoric arousal model (Solberg et al., 2016). Therefore, for some analyses, the symptoms were grouped in to five subfactors: Intrusions/Re-experiencing (items 1-5), Avoidance (items 6–7), Emotional numbing (items 8–12), Dysphoric arousal (items 13–15), and Anxious arosual (items 16–17).

We constructed a severity of direct exposure value by counting participants' number of direct exposure incidents. Respondents were asked whether they had (a) witnessed people who were dead or dying; (b) witnessed people who were seriously injured; and (c) whether they themselves had been physically injured. The response for each item was coded as 0 or 1 and added together to create a severity of direct exposure value that ranged from 0 to 3.

We also constructed a severity of indirect exposure value by assessing participants' number of indirect exposure incidents. The items assessed whether the respondents (a) lost a close colleague, (b) experienced that a close colleague was injured, and/or (c) experienced that their office was damaged due to the bomb attack. The response for each item was coded as 0 or 1 and added together to create a severity of indirect exposure value that ranged from 0 to 3. Furthermore, we also added all exposure variables together, creating a total exposure value that ranged from 0 to 6.

### Analyses

#### Gender differences in levels of symptoms

**Figure 1:**
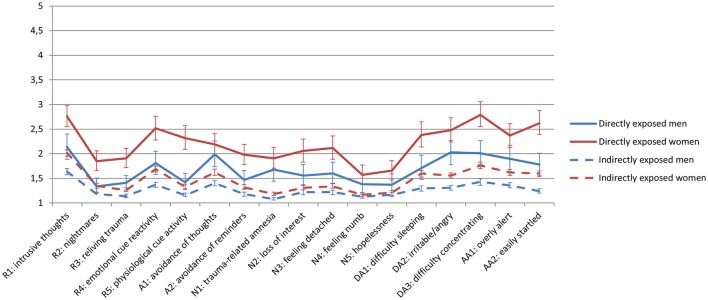
Mean levels with 95% CI of individual symptoms of posttraumatic stress in directly and indirectly exposed women and men 10 months after a terrorist attack.

In order to test gender differences in levels of demographic variables and exposure, we conducted chi square tests and *t*-tests for the directly and indirectly exposed individuals separately. We also used a bootstrap procedure (*n* = 1,000 samples) to estimate means and standard deviations with 95% CI of individual posttraumatic stress symptoms. We computed effect sizes by calculating the mean difference between women and men, and then dividing the result by the pooled standard deviation. An effect size of 0.2 is interpreted as “small,” effect size of 0.5 “medium” and 0.8 as “large” (Cohen, 1992). For the effect size for the chi square test, odds ratio was used. These analyses were conducted in IBM SPSS Statistics 24, and Excel 2016 was used for creating Figure 1.

#### Gender differences in relationships between symptoms

To examine the relationships between symptoms, we used network analyses. Due to the rather low prevalence of posttraumatic stress symptoms, especially in men, it was not meaningful to compute networks of symptoms in each gender, for directly and indirectly exposed groups separately. Therefore, for the network analyses, we excluded participants with low levels of posttraumatic stress symptoms, defined as sum of PCL < 30 (U. S. Department of Veteran Affairs, 2012), resulting in a sample of *n* = 375 individuals (23% of the total sample, 270 women and 105 men). Among these were 98 individuals directly exposed and 277 were indirectly exposed to the terrorist attack. Each of the DSM-IV PTSD symptoms were represented by a node in the network, and the strength of the associations between the symptoms (nodes) were represented by an edge between the nodes.

In line with the most recent methodological papers on network analysis used on psychological constructs such as PTSD (Epskamp et al., 2017; Costantini et al., in press; Fried et al., in press) we first estimated regularized partial correlation networks for each gender via the EstimateNetwork function of the R-package bootnet. The resulting estimates of edges can be interpreted as the correlation between two nodes after controlling for all other nodes in the network (partialling out common variance between nodes). Next, we employed the Fused Graphical Lasso (FGL; Danaher et al., 2014), using information criterion and otherwise default settings in the R-package EstimateGroupNetwork. This allows for jointly estimation of networks. Our analytic strategy generally followed the analytic strategy and R code provided by Fried et al. (in press).

##### Interconnectedness

We computed the centrality of each symptom in the network (Opsahl et al., 2010). Higher centrality of a symptom indicates a stronger association with other symptoms in the network (Borsboom and Cramer, 2013; Epskamp et al., 2017). Measures of centrality are relative metrics of interconnectedness. Three common types of centrality were assessed: strength, closeness, and betweenness. The strength of a node indicates the mean magnitude of the correlations of each edge linked to the node. It provides a measure of how strongly a node is directly connected to other nodes in the network. The closeness of a node takes the inverse of the sum of all shortest paths between a node and all other nodes in the network, investigating how strongly a node is indirectly connected to other nodes in the network. The betweenness of a node indicates the number of times that node lies on the shortest path between two other nodes. It can be interpreted as how central the node is in connecting other nodes.

In addition, we estimated predictability, which is an absolute metric of interconnectedness. Using the R-package mgm, we estimated shared variance of each node with all of its neighbors (Haslbeck and Fried, 2017). This is often termed predictability and quantifies how much influence the connections from the neighbor nodes can have on each node, assuming that all connections go toward this node. Thus, predictability is a value for the upper bound of shared variance. As the measure of predictability we used R square.

##### Robustness of networks

To ensure that the estimated networks were robust enough for interpretation, we followed the recommendations by Epskamp et al. (2017) and performed robustness analyses using the R-package bootnet. Bootstrapping procedures were used to examine two robustness-related issues: (1) the robustness of the edge weights; and (2) the robustness of the measures of node centrality. We assessed the variability of edge weights and centrality by estimating confidence intervals (CI) in which 95% of the cases contain the true value of the parameter (bootstrapped samples = 1,000). The network stability can be quantified by using the correlation stability (CS) coefficient. This measure quantifies the maximum proportion of cases that can be dropped to retain, with 95% certainty, a correlation with the original centrality of higher than (by default) 0.7. Based on a simulation study, Epskamp, Borsboom, and Fried suggest that to interpret centrality differences the CS-coefficient should preferably be above 0.50 (Epskamp et al., 2017).

##### Network comparison

To test differences between connectivity across the networks for men and women, we utilized the R package NetworkComparisonTest (NCT). With this package, it is possible to assess the difference between two networks based on invariance measures such as network structure invariance, global strength invariance, and edge invariance (van Borkulo, 2016; van Borkulo et al., 2017). In order to increase sensitivity gamma was set to 0, otherwise default settings were used.

## Results

### Gender differences in symptom levels

There were more women than men both in the direct and the indirect exposed subsamples (see Table 1). The severities of direct, indirect or total exposure among the directly exposed were not significantly different across gender. Similarly, the severity of indirect exposure among the indirectly exposed was not significantly different across gender.

**Table 1:** Characteristics of the sample.

	**Directly exposed (*****n*** **= 190)**						**Indirectly exposed (*****n*** **= 1615)**					
	**Women (*****n*** **= 117)**		**Men (*****n*** **= 73)**		**Diff**		**Women (*****n*** **= 918)**		**Men (*****n*** **= 697)**		**Diff**	
	**Mean/%**	**SD/*n***	**Mean/%**	**SD/n**	**F/chisq**	***p***	**Mean/%**	**SD/*n***	**Mean/%**	**SD/n**	**F/chisq**	***p***
**DEMOGRAPHICS**												
Age (years) M ± SD	44.5	1.2	45.1	1.2	0.140	0.709	44.4	1.06	46.5	1.10	15.993	<0.000
Education												
Low	11.1	13	8.2	6			13.2	121	8.0	56		
Mid	29.9	35	24.7	18	1.302	0.555	24.9	228	22.5	157	13.942	0.001
High	59.0	69	67.1	49			61.9	568	69.4	484		
**DIRECT EXPOSURE**												
Witnessed dead/dying people	31.0	36	37.5	27	0.833	0.361						
Witnessed seriously injured people	61.7	71	70.8	51	1.615	0.204						
Was injured	26.5	31	24.7	18	0.079	0.778						
**INDIRECT EXPOSURE**												
Injured close colleague	57.3	67	50.7	37	0.786	0.375	48.2	441	46.2	321	0.674	0.412
Dead close colleague	22.2	26	16.4	12	0.940	0.332	13.8	126	12.6	87	0.504	0.478
Office damage	64.7	75	72.6	53	1.295	0.255	54.6	501	50.9	355	2.110	0.146
**SUM SEVERITY OF EXPOSURE**	***M***	***SD***	***M***	***SD***	***t***	***P***						
Sum severity of direct exposure (0–3)	1.18	0.96	1.32	0.86	0.965	0.327						
Sum severity of indirect exposure (0–3)	1.44	0.99	1.40	0.92	0.067	0.788	1.16	0.98	1.09	0.96	1.870	0.160
Total severity of exposure (0–6)	2.62	1.59	2.71	1.59	0.422	0.683						

As can be seen in Figure 1 women reported higher levels of most symptoms compared to men. Furthermore, Tables 2, 3 also indicated that the standard deviations were smaller in men than in women in both the directly and the indirectly exposed subsample (see Figures S1–S4 in Supplementary Materials for histograms). There is evidence of floor effects in all the subsamples. In the directly exposed subsample, women reported higher levels of re-experiencing, emotional numbing, dysphoric arousal, and anxious arousal. The values of Cohen's d indicate greatest effect for the symptoms of re-experiencing (Cohen's *d* = 0.76), anxious arousal (Cohen's *d* = 0.58) and dysphoric arousal (Cohen's *d* = 0.58). These effects can be regarded as being between medium and large size. Similar differences were found in the indirectly exposed subsample, but of smaller magnitude. The effect size of the gender differences in sum of all the posttraumatic stress symptoms was medium to large (Cohen's d was 0.65 for the directly exposed, and 0.41 for the indirectly exposed subsample). Whereas the percentage of directly exposed women fulfilling the criteria of probable PTSD was 32%, only 12% of the men fulfilled the criteria of PTSD.

**Table 2:** Distributions of posttraumatic symptom scores in directly exposed individuals.

	**Women (*****n*** **= 117)**				**Men (*****n*** **= 73)**				***d***
	**Mean**	***SD***	**Mean 95% CI**	**SD 95% CI**	**Mean**	***SD***	**Mean 95% CI**	**SD 95% CI**	
**INDIVIDUAL SYMPTOMS OF PTSD**									
R1: intrusive thoughts	2.76	2.22	2.54–2.98	1.10–1.33	2.14	1.10	1.88–2.38	0.97–1.20	0.54
R2: nightmares	1.85	1.12	1.65–2.05	0.93–1.20	1.34	0.67	1.20–1.50	0.45–0.87	0.57
R3: reliving trauma	1.91	1.07	1.71–2.12	0.92–1.20	1.41	0.68	1.27–1.57	0.51–0.83	0.56
R4: emotional cue reactivity	2.52	1.28	2.29–2.75	1.14–1.40	1.81	1.01	1.59–2.04	0.82–1.17	0.62
R5: physiological cue activity	2.32	1.32	2.08–2.56	1.18–1.44	1.42	0.72	1.26–1.59	0.52–0.90	0.87
A1: avoidance of thoughts	2.19	1.25	1.93–2.42	1.09–1.40	1.99	1.12	1.74–2.22	0.96–1.27	0.17
A2: avoidance of reminders	1.98	1.17	1.76–2.19	1.02–1.30	1.47	0.82	1.30–1.65	0.62–0.97	0.52
N1: trauma-related amnesia	1.91	1.15	1.71–2.14	0.96–1.32	1.68	1.05	1.45–1.94	0.83–1.23	0.21
N2: loss of interest	2.06	1.29	1.84–2.29	1.22–1.43	1.56	0.91	1.37–1.76	0.67–1.12	0.45
N3: feeling detached	2.12	1.29	1.88–2.35	1.13–1.42	1.60	0.97	1.39–1.82	0.73–1.16	0.46
N4: feeling numb	1.57	1.04	1.40–1.76	0.83–1.21	1.38	0.83	1.22–1.57	0.57–1.02	0.20
N5: hopelessness	1.66	1.10	1.47–1.87	0.89–1.28	1.37	0.74	1.20–1.54	0.53–0.89	0.31
DA1: difficulty sleeping	2.38	1.42	2.10–2.67	1.28–1.54	1.71	1.10	1.48–1.95	0.86–1.27	0.53
DA2: irritable/angry	2.48	1.34	2.23–2.72	1.21–1.45	2.03	1.05	1.79–2.27	0.92–1.16	0.38
DA3: difficulty concentrating	2.79	1.39	2.53–3.05	1.27–1.49	2.01	1.07	1.77–2.25	0.87–1.23	0.63
AA1: overly alert	2.37	1.29	2.13–2.57	1.16–1.40	1.90	1.14	1.66–2.17	0.96–1.29	0.38
AA2: easily startled	2.62	1.38	2.37–2.87	1.25–1.49	1.78	0.99	1.57–2.00	0.81–1.13	0.71
**CLUSTERS OF PTSD**									
Re-experiencing	2.27	1.02	2.09–2.46	0.89–1.12	1.62	0.68	1.47–1.78	0.56–0.78	0.76
Avoidance	2.09	1.10	1.88–2.28	0.95–1.23	1.73	0.85	1.55–1.93	0.69–1.01	0.37
Emotional numbing	1.87	0.93	1.70–2.03	0.76–1.07	1.52	0.67	1.37–1.68	0.51–0.82	0.43
Dysphoric arousal	2.55	1.26	2.33–2.78	1.13–1.36	1.92	0.92	1.70–2.14	0.75–1.07	0.58
Anxious arousal	2.50	1.26	2.26–2.72	1.14–1.36	1.84	1.00	1.62–2.07	0.81–1.14	0.58
**OVERALL PTSD**									
Posttraumatic stress sum	37.50	16.02	34.62–40.41	13.81–18.02	28.62	11.21	26.13–31.17	9.09–13.06	0.65
	%	*n*			%	*n*			OR
Fulfilling criteria for PTSD	31.6	37			12.3	9			3.29

**Table 3:** Distributions of posttraumatic symptom scores in indirectly exposed women and men.

	**Women (*****n*** **= 918)**				**Men (*****n*** **= 697)**				***d***
	**Mean**	***SD***	**Mean 95% CI**	**SD 95% CI**	**Mean**	***SD***	**Mean 95% CI**	**SD 95% CI**	
**INDIVIDUAL SYMPTOMS OF PTSD**									
R1: intrusive thoughts	2.02	1.00	1.95–2.08	0.95–1.05	1.64	0.89	1.57–1.70	0.82–0.95	0.40
R2: nightmares	1.35	0.74	1.30–1.40	0.67–0.80	1.18	0.56	1,14–1.22	0.47–0.64	0.26
R3: reliving trauma	1.26	0.64	1.23–1.31	0.57–0.71	1.14	0.47	1.11–1.18	0.39–0.55	0.23
R4: emotional cue reactivity	1.69	0.92	1.63–1.75	0.86–0.97	1.37	0.71	1.32–1.42	0.64–0.77	0.39
R5: physiological cue activity	1.33	0.74	1.29–1.38	0.66–0.81	1.16	0.48	1.13–1.20	0.41–0.56	0.28
A1: avoidance of thoughts	1.62	0.95	1.56–1.69	0.89–1.02	1.40	0.78	1.35–1.46	0.71–0.84	0.26
A2: avoidance of reminders	1.32	0.77	1.28–1.37	0.69–0.84	1.18	0.57	1.14–1.22	0.47–0.65	0.21
N1: trauma-related amnesia	1.18	0.54	1.15–1.22	0.47–0.61	1.08	0.34	1.06–1.11	0.27–0.40	0.22
N2: loss of interest	1.31	0.71	1.27–1.36	0.64–0.78	1.22	0.61	1.17–1.27	0.52–0.69	0.14
N3: feeling detached	1.34	0.75	1.30–1.39	0.68–0.81	1.22	0.60	1.17–1.26	0.51–0.67	0.19
N4: feeling numb	1.17	0.52	1.13–1.20	0.45–0.58	1.13	0.44	1.10–1.17	0.36–0.50	0.07
N5: hopelessness	1.21	0.63	1.16–1.25	0.53–0.70	1.17	0.54	1.13–1.21	0.45–0.63	0.07
DA1: difficulty sleeping	1.60	1.03	1.53–1.66	0.96–1.09	1.30	0.73	1.24–1.35	0.63–0.81	0.34
DA2: irritable/angry	1.56	0.86	1.51–1.61	0.80–0.92	1.31	0.69	1.26–1.36	0.61–0.76	0.32
DA3: difficulty concentrating	1.76	1.00	1.70–1.83	0.94–1.06	1.43	0.80	1.36–1.49	0.73–0.87	0.37
AA1: overly alert	1.62	0.92	1.56–1.68	0.85–0.97	1.36	0.72	1.31–1.41	0.64–0.79	0.32
AA2: easily startled	1.60	0.88	1.54–1.65	0.82–0.94	1.24	0.61	1.20–1.29	0.53–0.69	0.47
**CLUSTERS OF PTSD**									
Re-experiencing	1.53	0.63	1.49–1.57	0.58–0.68	1.30	0.49	1.26–1.33	0.43–0.55	0.41
Avoidance	1.47	0.77	1.42–1.52	0.70–0.83	1.29	0.59	1.25–1.34	0.52–0.66	0.27
Emotional numbing	1.24	0.50	1.21–1.27	0.44–0.55	1.16	0.40	1.13–1.19	0.33–0.45	0.17
Dysphoric arousal	1.64	0.85	1.58–1.69	0.79–0.89	1.35	0.65	1.30–1.39	0.57–0.72	0.39
Anxious arousal	1.61	0.84	1.55–1.66	0.77–0.89	1.30	0.62	1.26–1.35	0.54–0.69	0.42
**OVERALL PTSD**									
Posttraumatic stress sum	24.94	9.27	24.36–25.51	8.51–9.98	21.53	7.39	20.97–22.06	6.40–8.31	0.41
	**%**	*n*			%	*n*			OR
Fulfilling criteria for PTSD	4.6	42			2.6	18			1.81

### Gender differences in relationships between symptoms

Table 4 show the distributions of posttraumatic stress symptoms in women and men with PCL sum score equal to or greater than 30. In these subsamples, there is no evidence of floor effects (see also Figures S5, S6 in the Supplementary Materials for histograms). As the overlapping confidence intervals of means and standard deviations indicate, there were few differences in level and variability of symptoms across gender. This is also reflected in the Pearson correlation between the means, which was 0.94 (*p* < 0.001). However, women reported significantly higher levels of two symptoms; physiological cue activity and feeling easily startled. The average symptom severity was 2.4 for women and 2.3 for men (range 1–5). The Pearson correlations between symptom means and symptom deviations were 0.35 (*p* = 0.161) for women and 0.36 (*p* = 0.153) for men, which do not indicate ceiling or floor effects.

**Table 4:** Distributions of posttraumatic symptom scores in women and men with sum of PCL ≥ 30.

	**Women (*****n*** **= 270)**				**Men (*****n*** **= 105)**				***d***
	**Mean**	***SD***	**Mean 95% CI**	**SD 95% CI**	**Mean**	***SD***	**Mean 95% CI**	**SD 95% CI**	
R1: intrusive thoughts	3.06	1.09	2.93–3.20	1.02–1.16	3.00	1.07	2.81–3.21	0.94–1.18	0.06
R2: nightmares	2.13	1.12	2.00–2.27	1.03–1.20	1.96	1.05	1.78–2.16	0.87–1.19	0.16
R3: reliving trauma	1.98	1.05	1.85–2.10	0.96–1.14	1.82	0.94	1.64–2.01	0.76–1.10	0.16
R4: emotional cue reactivity	2.81	1.15	2.66–2.95	1.08–1.23	2.58	1.02	2.40–2.77	0.98–1.12	0.21
R5: physiological cue activity	2.31	1.22	2.16–2.45	1.12–1.31	1.93	0.91	1.76–2.11	0.77–1.04	0.36
A1: avoidance of thoughts	2.60	1.22	2.46–2.76	1.14–1.29	2.59	1.12	2.36–2.81	1.01–1.24	0.01
A2: avoidance of reminders	2.20	1.22	2.06–2.36	1.12–1.32	2.00	1.07	1.81–2.20	0.98–1.21	0.17
N1: trauma-related amnesia	1.79	1.04	1.67–1.92	0.92–1.15	1.63	0.93	1.46–1.82	0.76–1.09	0.16
N2: loss of interest	2.28	1.15	2.14–2.42	1.07–1.24	2.34	1.06	2.14–2.55	0.93–1.18	0.05
N3: feeling detached	2.36	1.19	2.22–2.50	1.10–1.27	2.27	1.10	2.05–2.47	0.98–1.21	0.08
N4: feeling numb	1.70	0.99	1.59–1.82	0.88–1.09	1.82	0.96	1.65–2.00	0.83–1.06	0.12
N5: hopelessness	1.87	1.13	1.74–2.01	1.00–1.24	1.98	1.06	1.78–2.17	0.88–1.19	0.10
DA1: difficulty sleeping	2.84	1.31	2.68–3.00	1.23–1.38	2.55	1.25	2.33–2.80	1.10–1.37	0.23
DA2: irritable/angry	2.73	1.13	2.58–2.87	1.05–1.21	2.64	1.00	2.43–2.82	0.88–1.10	0.08
DA3: difficulty concentrating	3.21	1.07	3.09–3.35	1.00–1.15	2.99	0.92	2.82–3.17	0.81–1.02	0.22
AA1: overly alert	2.67	1.15	2.54–2.81	1.06–1.22	2.66	1.14	2.44–2.89	1.01–1.26	0.01
AA2: easily startled	1.82	1.16	2.68–2.96	1.07–1.23	2.45	1.04	2.26–2.66	0.91–1.17	0.57

**Figure 2:**
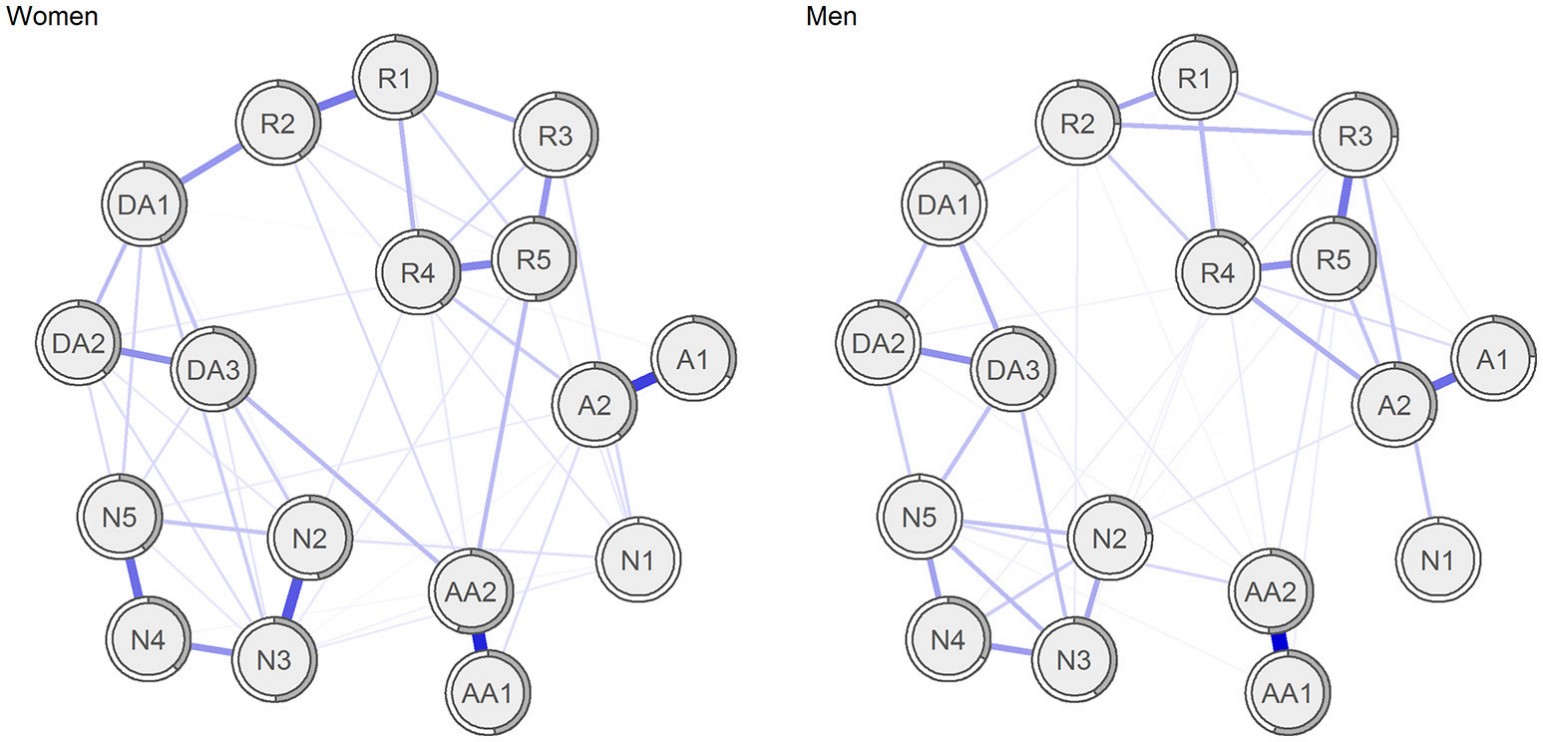
Networks of the symptoms of posttraumatic stress in women and men with sum of PCL ≥ 30.

Figure 2 shows the jointly estimated networks for women and men. The 95% CI around the edge-weights for each network estimated separately can be viewed in the Supplementary Materials (see Figures S7, S8). The generally large bootstrapped CIs suggest that interpreting the order of the edges in the network should be done with caution. Many of the bootstrapped CIs overlap each other, suggesting that many edge-weights do not significantly differ from one another. However, several of the edge-weights were reliably stronger than most of the others. In each network there were (17 ^*^ 16) / 2 = 136 possible edges (after the glasso estimation, 122 and 108 were retained for women and men, respectively). Edge-weights significantly stronger than 80% of the possible edges (109 edges) will be reported. For both women and men, the edge-weight between feeling easily startled (AA1) and overly alert (AA2) was significantly stronger than virtually all the other edge-weights. In addition, in women, the edge weights between avoidance of thoughts (A1) and avoidance of reminders (A2), between feeling detached (N3) and loss of interest (N2), and between feeling numb (N4) and hopelessness (N5), between intrusive thoughts (R1) and nightmares (R2), between emotional cue reactivity (R4) and physiological cue activity (R5), between nightmares (R2) and sleeping difficulties (DA1) were stronger than 80% of the other possible edge weights (for details, see Figures S9, S10 in the Supplementary Materials).

**Figure 3:**
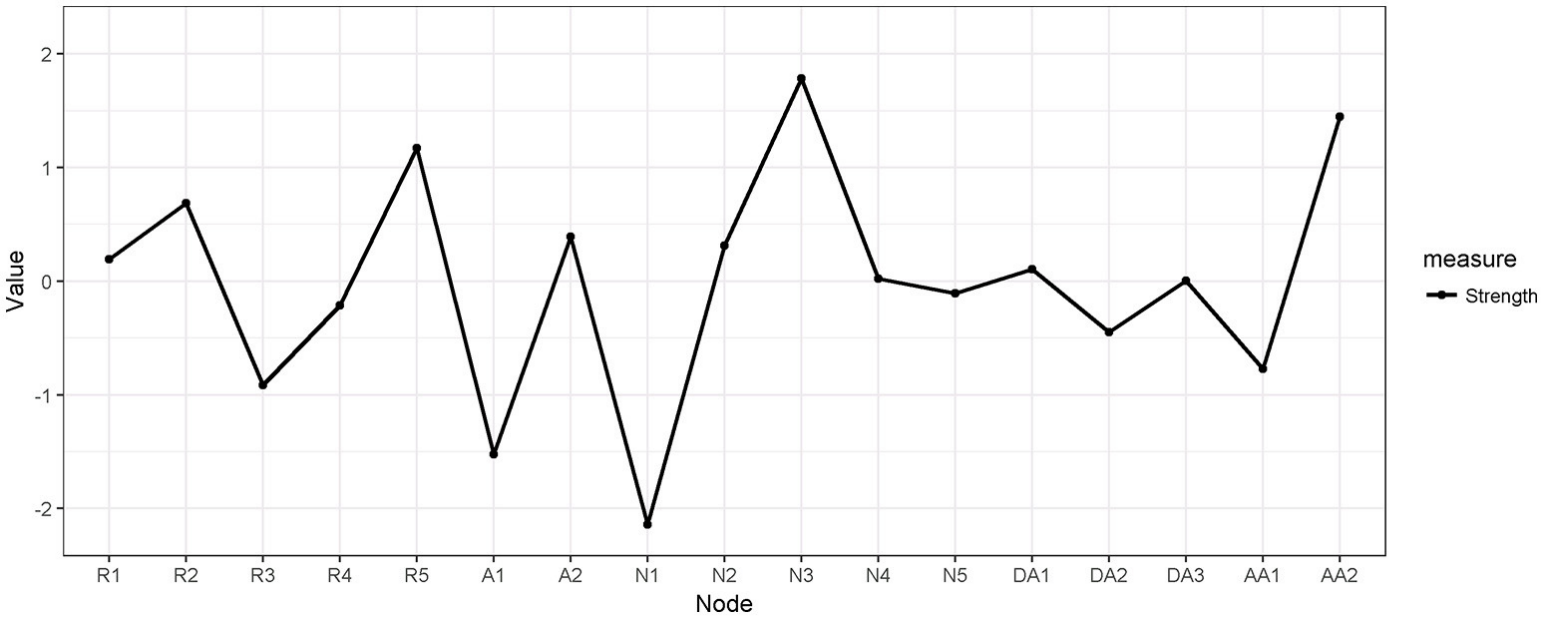
Estimates of node strength for the symptoms of posttraumatic stress in women with sum of PCL ≥ 30.

We also computed measures of node centrality (betweenness, closeness, and strength) for the separate networks, and computed correlation stability (CS) coefficients. The CS coefficients for betweenness were 0.12 and 0.00, for women and men, respectively. The corresponding CS coefficients for closeness were 0.28 and 0.00, and for strength 0.52 and 0.12. As the CS coefficients should exceed 0.50 to be interpretable, these values indicate that neither were reliably estimated in men. However, in women, node strength was sufficiently stable to be interpreted. For details, see Figures S11, S12 in the Supplementary Materials. Figure 3 presents the centrality plot of strength in women and indicates that feeling detached (N3) and easily startled (AA2) were the symptoms with highest strengths among women, although only significantly higher strength than eight and seven, respectively, of the other 16 symptoms (see Figure S13 in the Supplementary Materials). Furthermore, trauma-amnesia (N1) had significantly lower strength than 12 than the 16 other symptoms).

As differences in connection strength between symptoms may be caused by differences in variances, we tested whether the two-tailed Pearson correlations between node variance and node strength were significant. These correlations were *r*(15) = 0.34 (*p* = 0.17) for women and *r*(15) = −0.20 (*p* = 0.42) for men, which indicated that differential variability across symptoms did not pose a problem for interpreting a symptom's strength centrality (see Terluin et al., 2016).

On average, the proportion of explained variance (*R*^2^) explained by each node's neighbors was 0.43 in women and 0.37 in men. The values of for predictability for each node are presented in Table 5. Mirroring the values of node strengths, the anxious arousal symptoms (AA1 and AA2) had high predictability (*R*^2^ ranged between 0.50 and 0.63) whereas trauma-related amnesia (N1) had low predictability (*R*^2^ ranged between 0.02 and 0.15).

**Table 5:** Predictability (*R*^2^, the proportion of explained variance) of posttraumatic symptom scores in women and men with sum of PCL ≥ 30.

	**Women (*n* = 270)**	**Men (*n* = 105)**
R1: intrusive thoughts	0.43	0.24
R2: nightmares	0.43	0.23
R3: reliving trauma	0.34	0.48
R4: emotional cue reactivity	0.43	0.52
R5: physiological cue activity	0.51	0.54
A1: avoidance of thoughts	0.35	0.25
A2: avoidance of reminders	0.43	0.33
N1: trauma-related amnesia	0.15	0.02
N2: loss of interest	0.54	0.31
N3: feeling detached	0.59	0.39
N4: feeling numb	0.38	0.34
N5: hopelessness	0.41	0.47
DA1: difficulty sleeping	0.43	0.27
DA2: irritable/angry	0.42	0.31
DA3: difficulty concentrating	0.44	0.46
AA1: overly alert	0.50	0.57
AA2: easily startled	0.62	0.63
Mean	0.43	0.37

The Spearman rank correlation between the edge weights in the two networks was 0.56 (*p* < 0.001), indicating relatively strong similarities. We also tested formally whether the network structure was different for women compared to men. The network invariance test indicated no significant differences in the network topology (*M* = 0.23, *p* = 0.397), and no significant differences in global strength across networks (women: 7.32, men: 6.12, *s* = 1.20, *p* = 0.069). However, as the compared networks were based on rather small samples, which were also of different size, this finding might also be a result of low power, and should be interpreted with caution.

## Discussion

The present study investigated gender differences in the levels of posttraumatic stress symptoms and associations between symptoms 10 months after direct and indirect exposure to a terrorist attack. Despite very similar exposure to trauma, women generally reported higher levels of PTSD symptoms both after direct and indirect exposure. In line with our hypothesis, the largest magnitudes of gender differences were found for symptoms of re-experiencing, and anxious and dysphoric arousal. The gender differences for symptoms of avoidance and emotional numbing were smaller. Our results are in line with previous studies showing that women report higher levels of particularly re-experiencing and hyperarousal symptoms both after direct (Fullerton et al., 2001) and indirect (Stuber et al., 2006) exposure to trauma.

Whereas the severity of the symptoms according to exposure type differed across gender, individuals with posttraumatic stress symptoms reported similar profiles of posttraumatic symptoms. Furthermore, no significant gender differences in the networks of posttraumatic stress were found. This may not be surprising, given that most studies of factor structures of PTSD have found negligible differences across gender (Wang et al., 2013; Carragher et al., 2016; Frankfurt et al., 2016). Taken together, these findings suggest that the gender differences in prevalence of PTSD cannot be explained by differences in the processes of PTSD itself, but may rather be attributed to differences in PTSD-eliciting processes.

Given the same level of exposure, women reported higher levels of re-experiencing and anxious arousal. Furthermore, given similar levels of PTSD, women reported higher levels of physiological cue activity and feeling more easily startled. McNally et al. (2017) found that physiological cue activity was especially central in a directed acyclic graph network of PTSD symptoms, indicating that this symptom may act as one of the main driving forces of the posttraumatic stress symptomatology. This is in line with theories that argue that traumatic memories account for the relationship between trauma and other PTSD symptoms (Rubin et al., 2008), that the memories can contribute in maintaining a sense of current threat (Ehlers and Clark, 2000) and neurobiological accounts of PTSD as a stress-induced fear circuitry disorder (Shin and Handwerger, 2009).

Thus, higher proneness to re-experiencing symptoms such as physiological cue activity may explain at least some part of the gender differences in PTSD prevalence. Indeed, previous research has shown that there are important gender differences in emotional autobiographical memories (Pillemer et al., 2003). For example, men and women may remember different aspects of an emotional event. Experimental studies using aversive images and film clips in laboratory settings indicate that men show better voluntary memory of the gist of an emotional story, whereas women better remember the details of an emotional story (Cahill, 2003; Nielsen et al., 2013). Enhanced memory for details of a negative or traumatic event may in turn promote intrusiveness and persistence of these memories. Indeed, studies have also reported gender differences in involuntary memory, women have more intrusions after viewing emotional films compared to men (Ferree and Cahill, 2009). Behind these processes, there may be sex differences in the neuro-endocrinological response to stress (Merz and Wolf, 2017). Sex hormones are also associated with sensory strength and vividness of mental imagery (Wassell et al., 2015).

No prior network analyses of PTSD have explored potential gender differences. As in most other network studies, we found a strong associations between being overly alert and easily startled (McNally et al., 2015, 2017; Armour et al., 2017; Birkeland and Heir, 2017; Bryant et al., 2017; Spiller et al., 2017). Contrary to previous studies, we found that feeling easily startled was among the most central symptoms. This may be due to the nature of the traumatic event, as a bombing directed toward the workplace may occur at any time and without warning, which can trigger a state of anxious arousal. Trauma-related amnesia was not found to have central role in the symptom network. This is also one of the most consistent findings across the network studies (Armour et al., 2017; McNally et al., 2017) and is also consistent with findings from other studies (Geraerts and McNally, 2008; Armour et al., 2016), which suggest that trauma-related amnesia may not be regarded a core symptom of PTSD. In general, the current network studies on PTSD indicate that many of the associations between symptoms are similar across type of exposure, but that the most central symptoms of PTSD vary somewhat from study from study.

Among the strengths of this study is that we compared symptoms of PTSD in men and women who had experienced the same event, and that the time interval between the event and the measurement was the same for all respondents (ten months). In addition, our use of network analysis allowed for assessments of the network structure. One of the limitations is that we used self-report cross-sectional data, which do not allow for causal inferences. Low sample size has probably contributed to the moderate stability of the estimates, which made some of the results difficult to interpret. Replication with other community samples with larger sizes needs to be done. Although we did not find significant differences in variance in men compared to women, the direction of the non-significant variance effects were not consistent for men vs. women. Furthermore, even in the subsample of individuals with PCL ≥ 30, some symptoms were of low prevalence (e.g., trauma-related amnesia and feeling numb). Including more sensitive measurements of psychological processes that may play important roles in maintaining PTSD would be an improvement in future network analyses. There may also be gender differences in specific emotional, cognitive, or neurobiological correlates to posttraumatic stress that we have not assessed in the current study.

In conclusion, as women were more prone to re-experiencing symptoms in particular, gender differences in prevalence of PTSD may relate to gender differences in re-experiencing—the content and frequencies of the intrusive memories of the traumatic event. As we found no gender differences in symptom networks, the functional links among the symptoms of PTSD might be similar across gender. However, this finding may have resulted from low power due to the low sample size, especially of men with considerable levels of posttraumatic stress symptoms. Therefore, this hypothesis needs to be confirmed by additional studies exploring gender differences in networks of PTSD. However, the present results find no indication that the gender difference in prevalence of PTSD can be explained by differences in associations between symptoms. That women report higher levels of re-experiencing suggests that this gender difference may rather be attributed to differences in PTSD-eliciting processes such as encoding and memories of a traumatic event.

These findings suggest that interventions that reduce re-experiencing symptoms may be a potent way of helping both women and men to recover from traumatic experiences. Furthermore, that the networks were somewhat unstable indicate that power is an important issue also for network analyses. This also suggest that visual results from networks need to be interpreted with caution, and should be supplemented with stability analyses. In addition, further research that includes measures on how men and women remember traumatic events, as well as cope with symptoms of PTSD such as re-experiencing and anxious arousal will be useful in increasing the understanding of the gender differences in PTSD.

## Ethics statement

This study was carried out in accordance with the recommendations of the Norwegian Regional Committees for medical and health research ethics with written informed consent from all subjects. All subjects gave written informed consent in accordance with the Declaration of Helsinki. The protocol was approved by the REK sør-øst.

## Author contributions

MB, IB, ØS, and TH: conceived of the study, developed the study material and carried out data collection; MB: analyzed the data; All authors drafted the manuscript, and read and approved the final manuscript.

### Conflict of interest statement

The authors declare that the research was conducted in the absence of any commercial or financial relationships that could be construed as a potential conflict of interest.
