# Supplementary material for: Gender Differences in Posttraumatic Stress Symptoms after a Terrorist Attack: A Network Approach
Source: Front Psychol. 2017 Dec 1;8:2091. doi: 10.3389/fpsyg.2017.02091 (PMC5717368; doi:10.3389/fpsyg.2017.02091)
Supplement: Supplementary file 1 [file Presentation1.PDF]

Supplementary materials for:

Gender differences in posttraumatic stress symptoms after a terrorist attack: A network approach

Marianne Skogbrott Birkeland[1], Ines Blix[1], Øivind Solberg[1], & Trond Heir[1, 2]

Norwegian Centre for Violence and Traumatic Stress Studies (NKVTS)

Author Note

[1]Norwegian Centre for Violence and Traumatic Stress Studies, Norway

[2] Institute of Clinical Medicine, Faculty of Medicine, University of Oslo, Norway.

Correspondence concerning this article should be addressed to Marianne Skogbrott Birkeland, Norwegian Centre for Violence and Traumatic Stress Studies, P.O. Box 181 Nydalen, 0409 Oslo, Norway, marianne.s.birkeland@gmail.com

Supplementary information about the distributions

Histograms showing distributions of each symptom for directly exposed women and men can be viewed in Figures S1 and S2. Histograms for indirectly exposed women and men are shown in Figures S3 and S4. Figures S5 and S6 show histograms for women and men with mean PCL above cutoff (≥30). The data presented in Figures S5 and S6 is used for the network analyses.

Supplemental information about the robustness of networks

In order to assess robustness and stability of the networks, we followed the recommendations by Epskamp, Borsboom and Fried (Epskamp, Borsboom, & Fried, 2017). The R-package bootnet was used to assess the variability of edge weights and centrality measured by estimated confidence intervals (CI) where 95% of the cases will contain the true value of the parameter (bootstrapped samples = 1000).

The 95% CI around the edge weights for the networks of posttraumatic stress are displayed in Figures S7 and S8 for women and men, respectively. The generally large bootstrapped CIs suggest that interpreting the order of most edges in the network should be done carefully.

Figures S9 and S10 show the results from the bootstrapped difference tests ($\alpha = 0.05$) between edge-weights that were non-zero in the networks of posttraumatic stress clusters for women and men, respectively. In each network there were (17 * 16) / 2 =136 possible edges (after the glasso estimation, 122 and 108 were edges retained for women and men, respectively). Edge-weights significantly stronger than 80% of the possible edges (109) will be reported. For both women and men, the edge-weight between feeling easily startled (AA1) and overly alert (AA2) was significantly stronger than virtually all the other edge-weights. In addition, in women, the edge weights between avoidance of thoughts (A1) and avoidance of reminders (A2), between feeling detached (N3) and loss of interest (N2), and between feeling

numb (N4) and hopelessness (N5), between intrusive thoughts (R1) and nightmares (R2), between emotional cue reactivity (R4) and physiological cue activity (R5), between nightmares (R2) and sleeping difficulties (DA1) were stronger than 80 % of the other possible edge weights

The results from the person-dropping bootstrap of centrality measures are shown in Figures S11 and S12. As computations of correlation stability coefficient indicated, the measures of betweenness and closeness were not stable under subsetting cases, and strength was only stable among women (see also main paper). We concluded that the order of the node strength as interpretable among women with some caution.

The results from the bootstrapped difference tests ($\alpha = 0.05$) between values of node strength for women are shown in Figures S13. Feeling detached (N3) and easily startled (AA2) were the symptoms with highest strengths, although they showed only significantly higher strength than approximately half of the other symptoms. Furthermore, trauma-amnesia (N1) had significantly lower strength than 12 of the 16 other symptoms.

References

Epskamp, S., Borsboom, D., & Fried, E. (2017). Estimating Psychological Networks and their Accuracy: A Tutorial Paper. Retrieved from https://arxiv.org/abs/1604.08462

Supplemental Figure Captions

Figure S1.

Histograms showing distributions of each PTSD symptom for directly exposed women

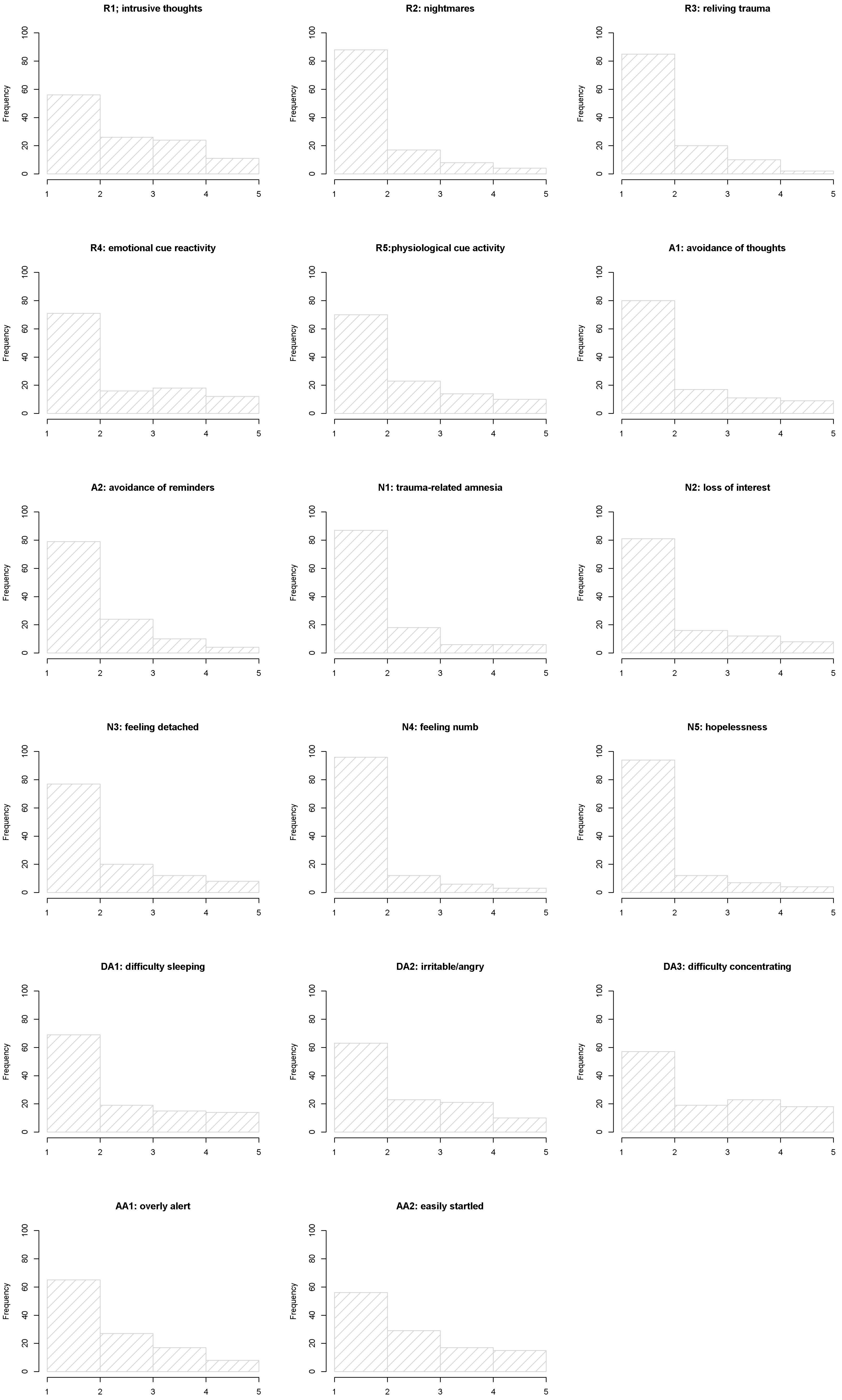
R1; intrusive thoughts
R2: nightmares
R3: reliving trauma
R4: emotional cue reactivity
R5:physiological cue activity
A1: avoidance of thoughts
A2: avoidance of reminders
N1: trauma-related amnesia
N2: loss of interest
N3: feeling detached
N4: feeling numb
N5: hopelessness
DA1: difficulty sleeping
DA2: irritable/angry
DA3: difficulty concentrating
AA1: overly alert
AA2: easily startled
Frequency
0 20 40 60 80 100
1 2 3 4 5

Figure S2.

Histograms showing distributions of each PTSD symptom for directly exposed men

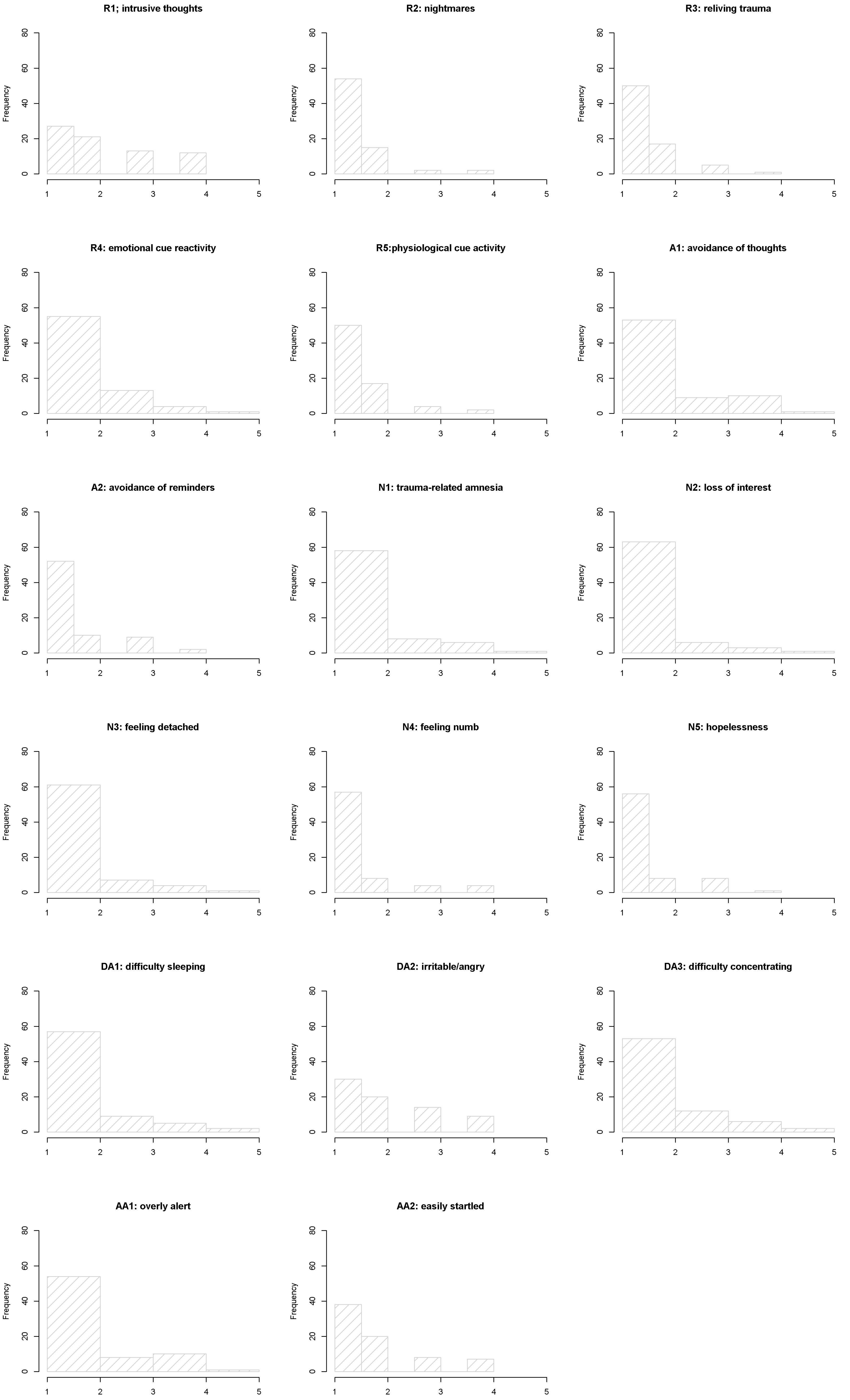
R1; intrusive thoughts
R2: nightmares
R3: reliving trauma
R4: emotional cue reactivity
R5:physiological cue activity
A1: avoidance of thoughts
A2: avoidance of reminders
N1: trauma-related amnesia
N2: loss of interest
N3: feeling detached
N4: feeling numb
N5: hopelessness
DA1: difficulty sleeping
DA2: irritable/angry
DA3: difficulty concentrating
AA1: overly alert
AA2: easily startled
Frequency
0
20
40
60
80
1
2
3
4
5

Figure S3.

Histograms showing distributions of each PTSD symptom for indirectly exposed women

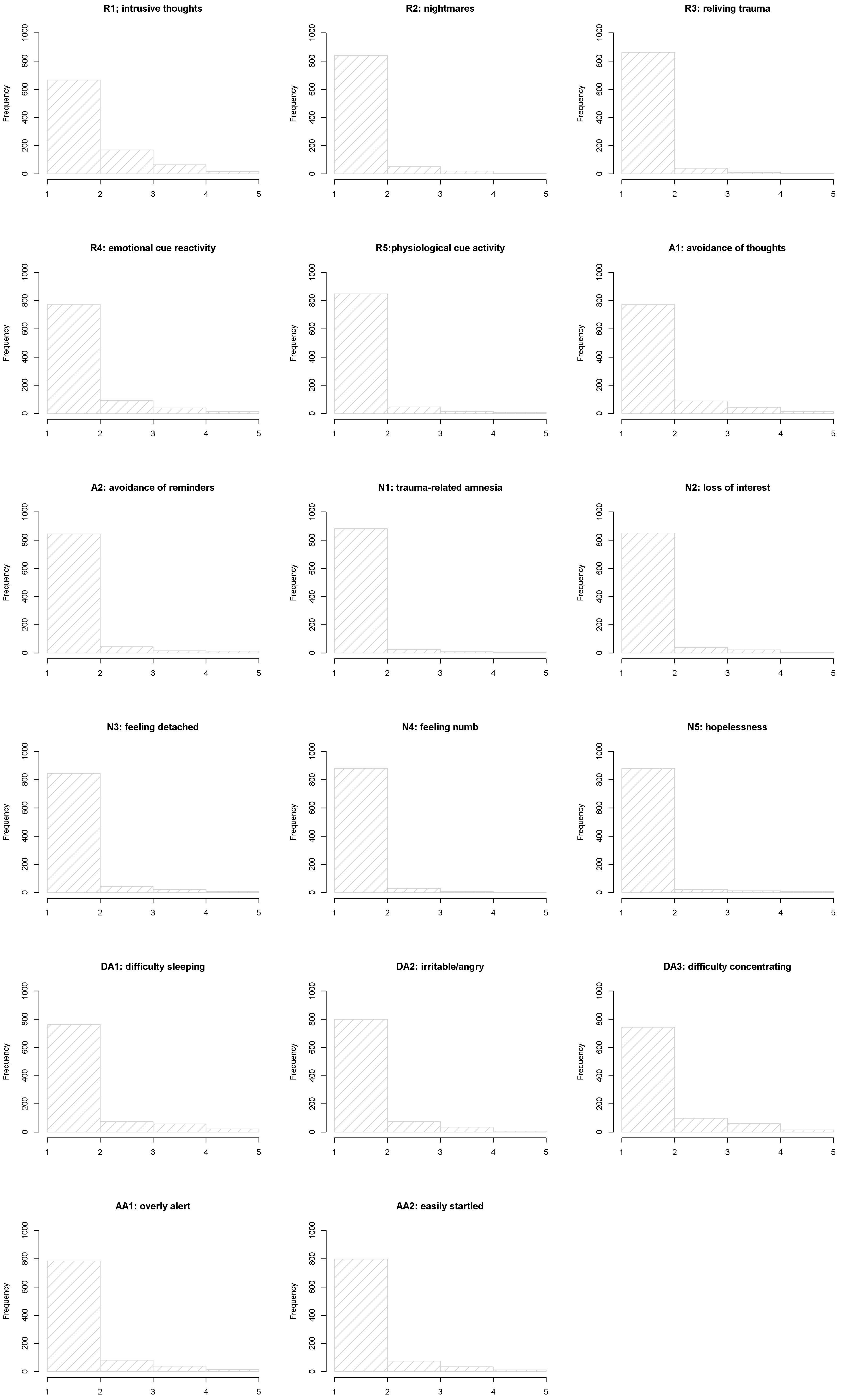

R1; intrusive thoughts
R2: nightmares
R3: reliving trauma
R4: emotional cue reactivity
R5:physiological cue activity
A1: avoidance of thoughts
A2: avoidance of reminders
N1: trauma-related amnesia
N2: loss of interest
N3: feeling detached
N4: feeling numb
N5: hopelessness
DA1: difficulty sleeping
DA2: irritable/angry
DA3: difficulty concentrating
AA1: overly alert
AA2: easily startled
Frequency
0
200
400
600
800
1000
1
2
3
4
5

Figure S4.

Histograms showing distributions of each PTSD symptom for indirectly exposed men

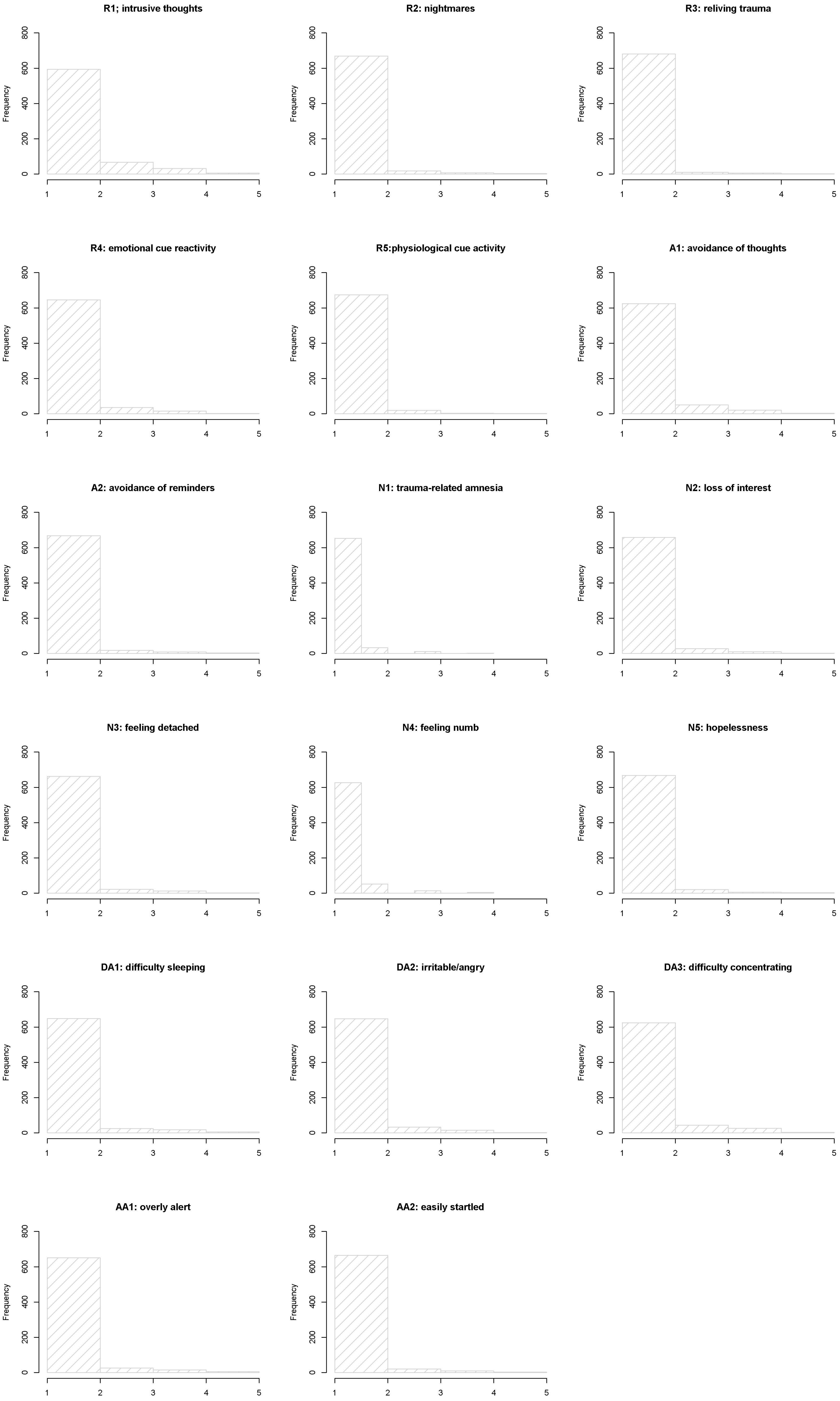

R1; intrusive thoughts
R2: nightmares
R3: reliving trauma
R4: emotional cue reactivity
R5:physiological cue activity
A1: avoidance of thoughts
A2: avoidance of reminders
N1: trauma-related amnesia
N2: loss of interest
N3: feeling detached
N4: feeling numb
N5: hopelessness
DA1: difficulty sleeping
DA2: irritable/angry
DA3: difficulty concentrating
AA1: overly alert
AA2: easily startled
Frequency
0
200
400
600
800
1
2
3
4
5

Figure S5.

Histograms showing distributions of each PTSD symptom for women with mean of PCL ≥30

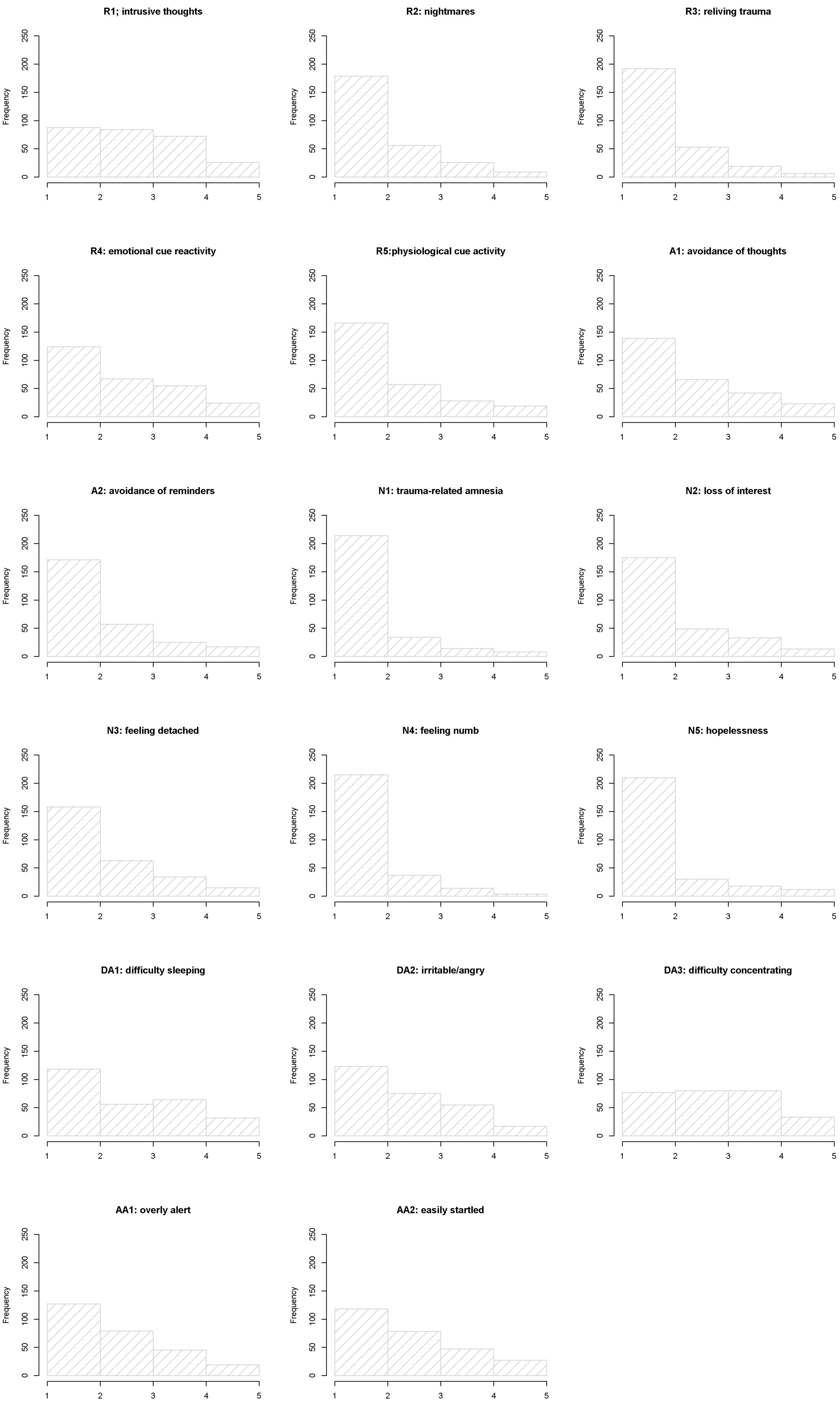
R1; intrusive thoughts
R2: nightmares
R3: reliving trauma
R4: emotional cue reactivity
R5:physiological cue activity
A1: avoidance of thoughts
A2: avoidance of reminders
N1: trauma-related amnesia
N2: loss of interest
N3: feeling detached
N4: feeling numb
N5: hopelessness
DA1: difficulty sleeping
DA2: irritable/angry
DA3: difficulty concentrating
AA1: overly alert
AA2: easily startled
Frequency
0
50
100
150
200
250
1
2
3
4
5

Figure S6.

Histograms showing distributions of each PTSD symptom for men with mean of PCL ≥30

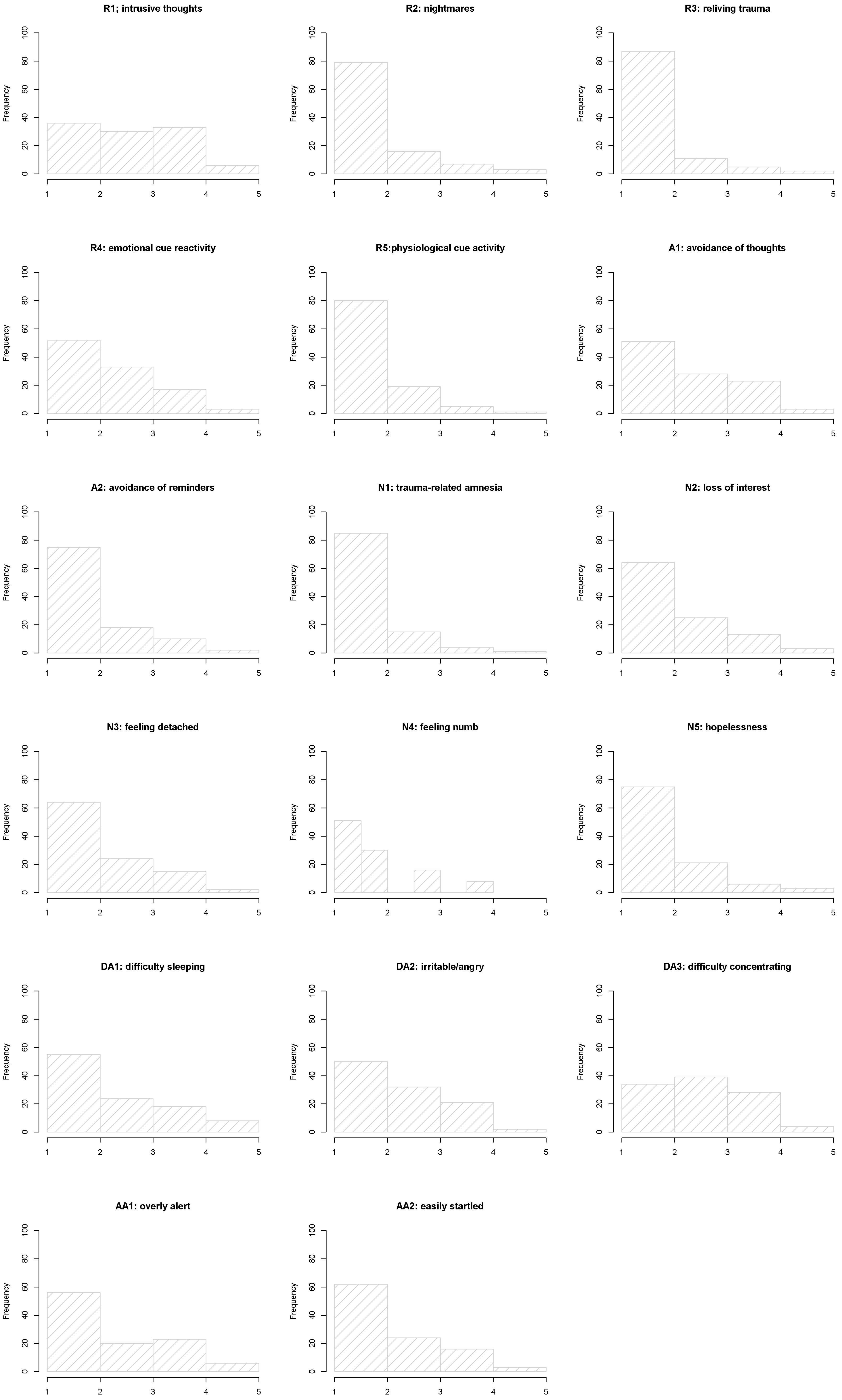
R1; intrusive thoughts
R2: nightmares
R3: reliving trauma
R4: emotional cue reactivity
R5:physiological cue activity
A1: avoidance of thoughts
A2: avoidance of reminders
N1: trauma-related amnesia
N2: loss of interest
N3: feeling detached
N4: feeling numb
N5: hopelessness
DA1: difficulty sleeping
DA2: irritable/angry
DA3: difficulty concentrating
AA1: overly alert
AA2: easily startled
Frequency
0
20
40
60
80
100
1
2
3
4
5

Figure S7.

Bootstrapped confidence intervals of estimated edge weights for the network of posttraumatic stress clusters for women. The red line indicates the edge weights and the gray area the 95% CIs.

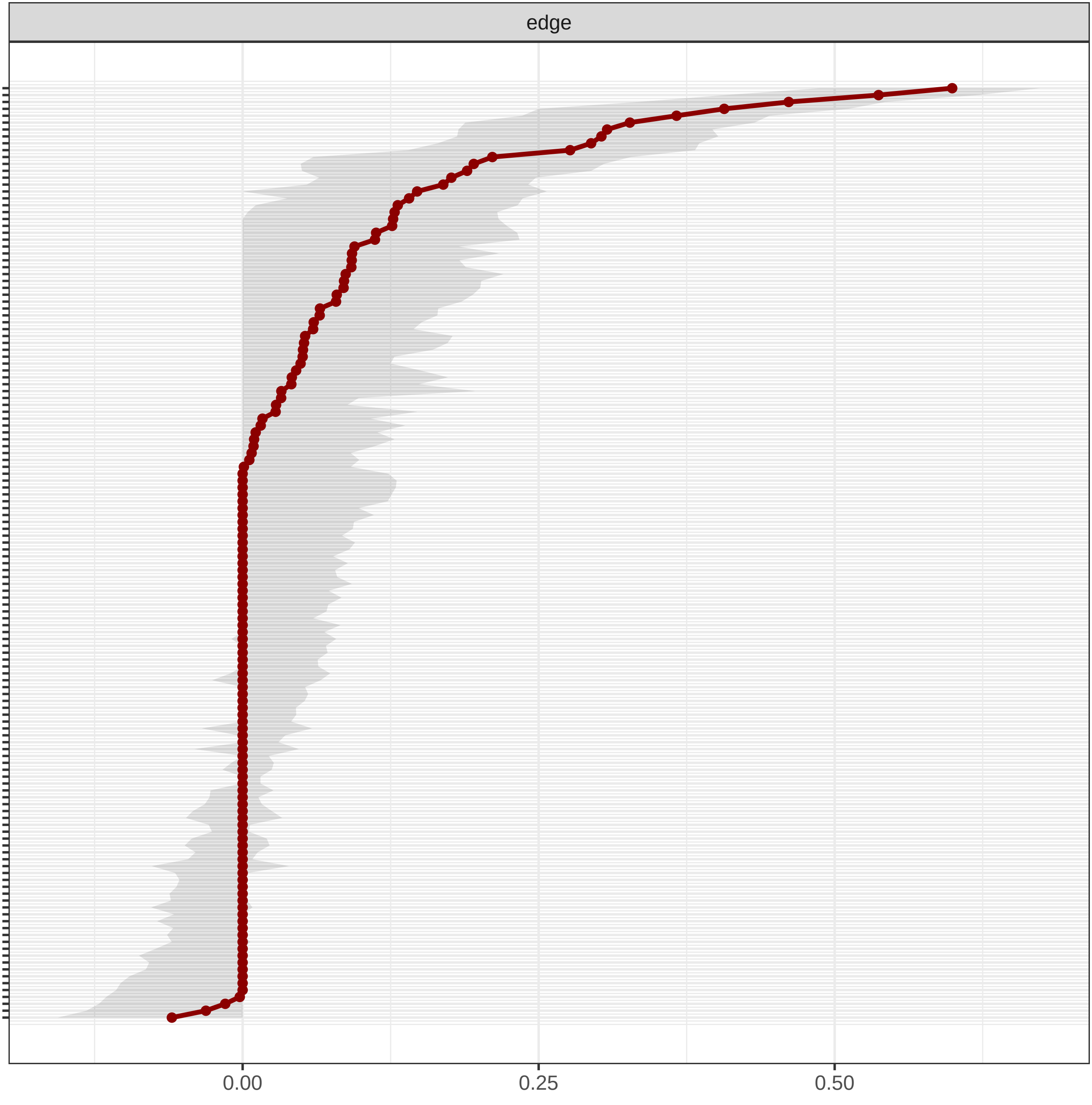

edge
0.00
0.25
0.50

Figure S8.

Bootstrapped confidence intervals of estimated edge weights for the glasso network of posttraumatic stress clusters for men. The red line indicates the edge weights and the gray area the 95% CIs.

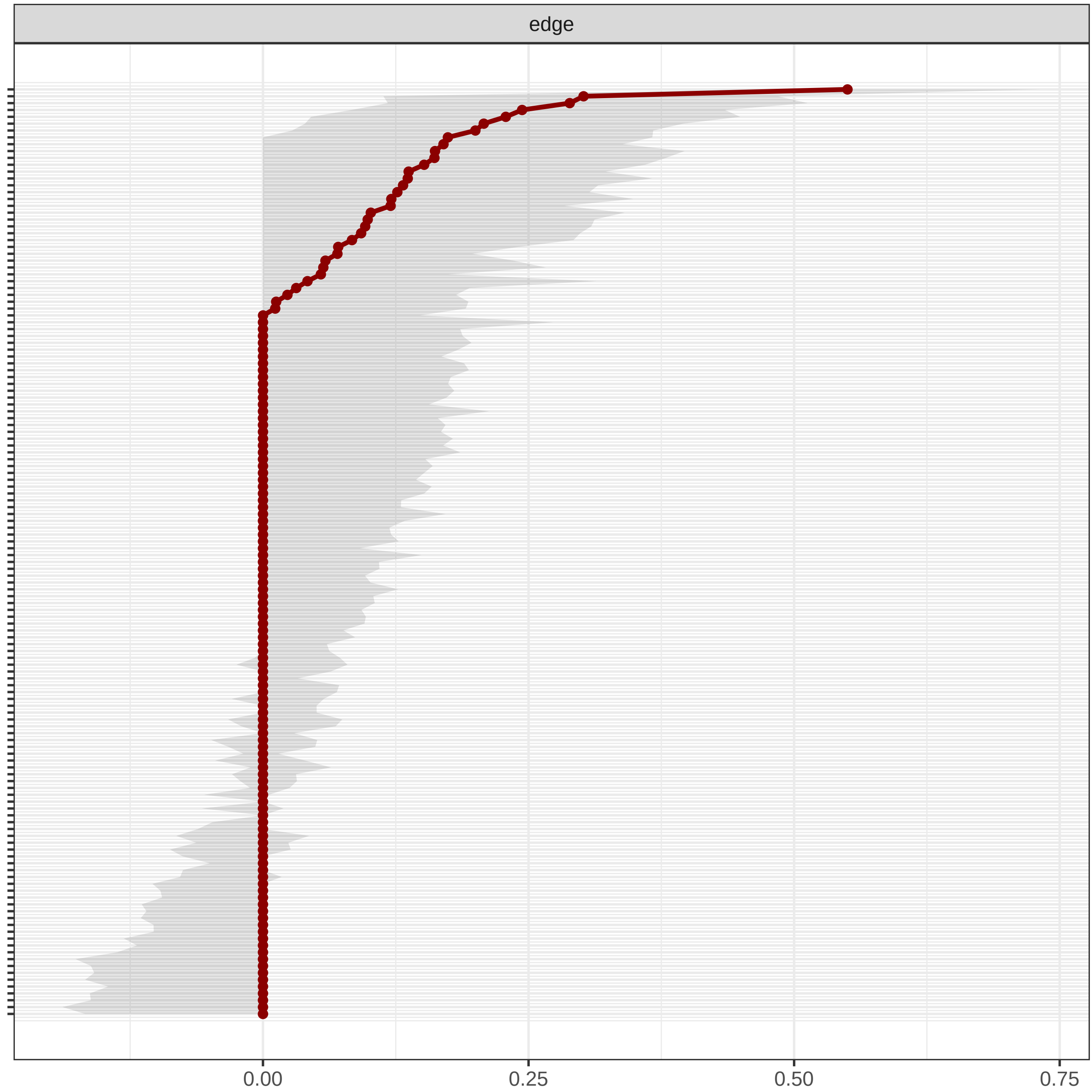

edge
0.00
0.25
0.50
0.75

Figure S9.

Bootstrapped difference tests (α = 0.05) between edge-weights that were non-zero in the networks of posttraumatic stress clusters for women. Gray boxes indicate edges that do not differ significantly from one-another and black boxes represent edges that do differ significantly from one-another. The different shades of blue in the edge-weight plot represent the magnitude of the edge-weights, and correspond to the shades of blue in Figure 2.

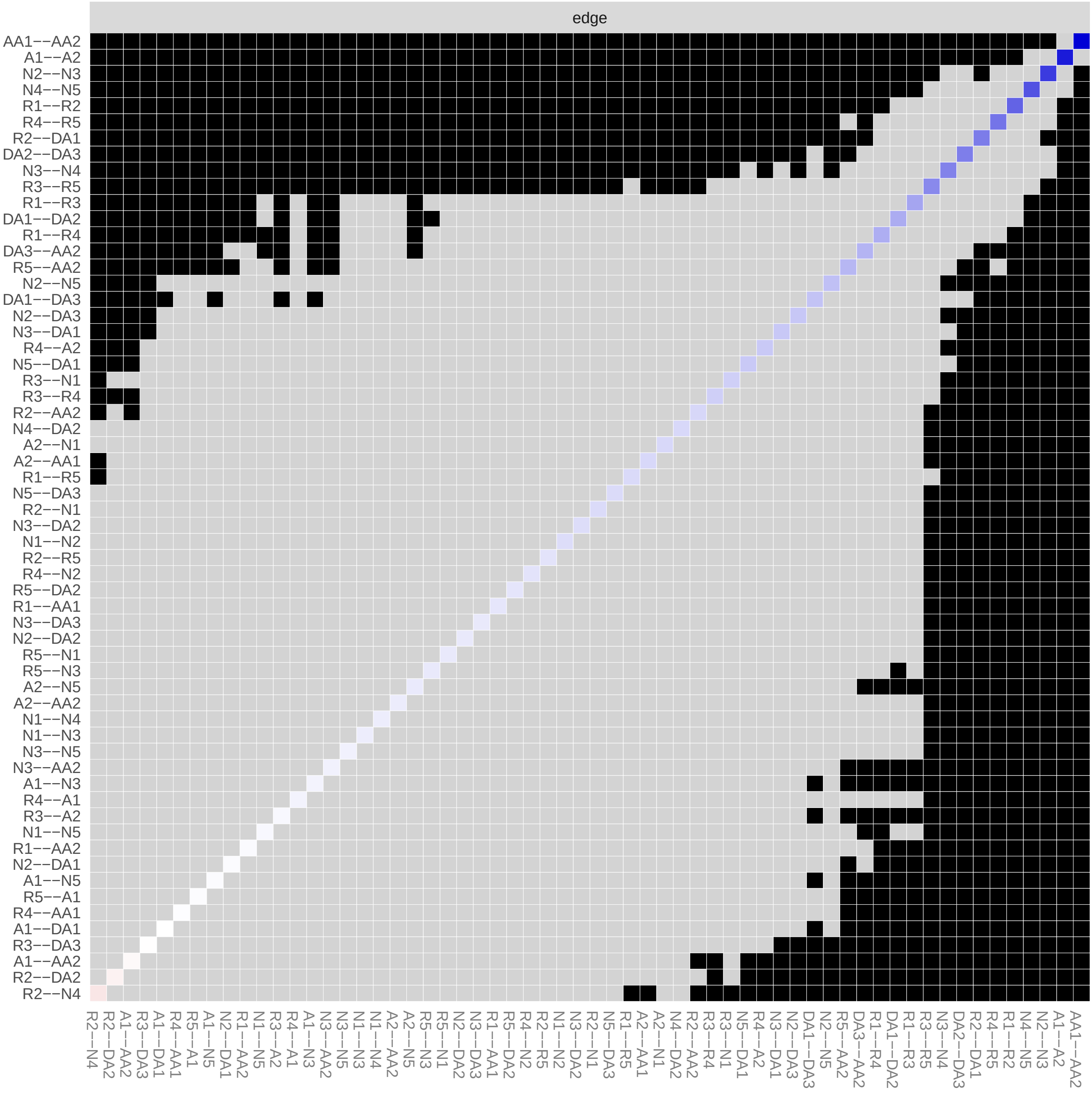

edge
AA1--AA2
A1--A2
N2--N3
N4--N5
R1--R2
R4--R5
R2--DA1
DA2--DA3
N3--N4
R3--R5
R1--R3
DA1--DA2
R1--R4
DA3--AA2
R5--AA2
N2--N5
DA1--DA3
N2--DA3
N3--DA1
R4--A2
N5--DA1
R3--N1
R3--R4
R2--AA2
N4--DA2
A2--N1
A2--AA1
R1--R5
N5--DA3
R2--N1
N3--DA2
N1--N2
R2--R5
R4--N2
R5--DA2
R1--AA1
N3--DA3
N2--DA2
R5--N1
R5--N3
A2--N5
A2--AA2
N1--N4
N1--N3
N3--N5
N3--AA2
A1--N3
R4--A1
R3--A2
N1--N5
R1--AA2
N2--DA1
A1--N5
R5--A1
R4--AA1
A1--DA1
R3--DA3
A1--AA2
R2--DA2
R2--N4

Figure S10.

Bootstrapped difference tests (α = 0.05) between edge-weights that were non-zero in the networks of posttraumatic stress clusters for men. Gray boxes indicate edges that do not differ significantly from one-another and black boxes represent edges that do differ significantly from one-another. The different shades of blue in the edge-weight plot represent the magnitude of the edge-weights, and correspond to the shades of blue in Figure 2.

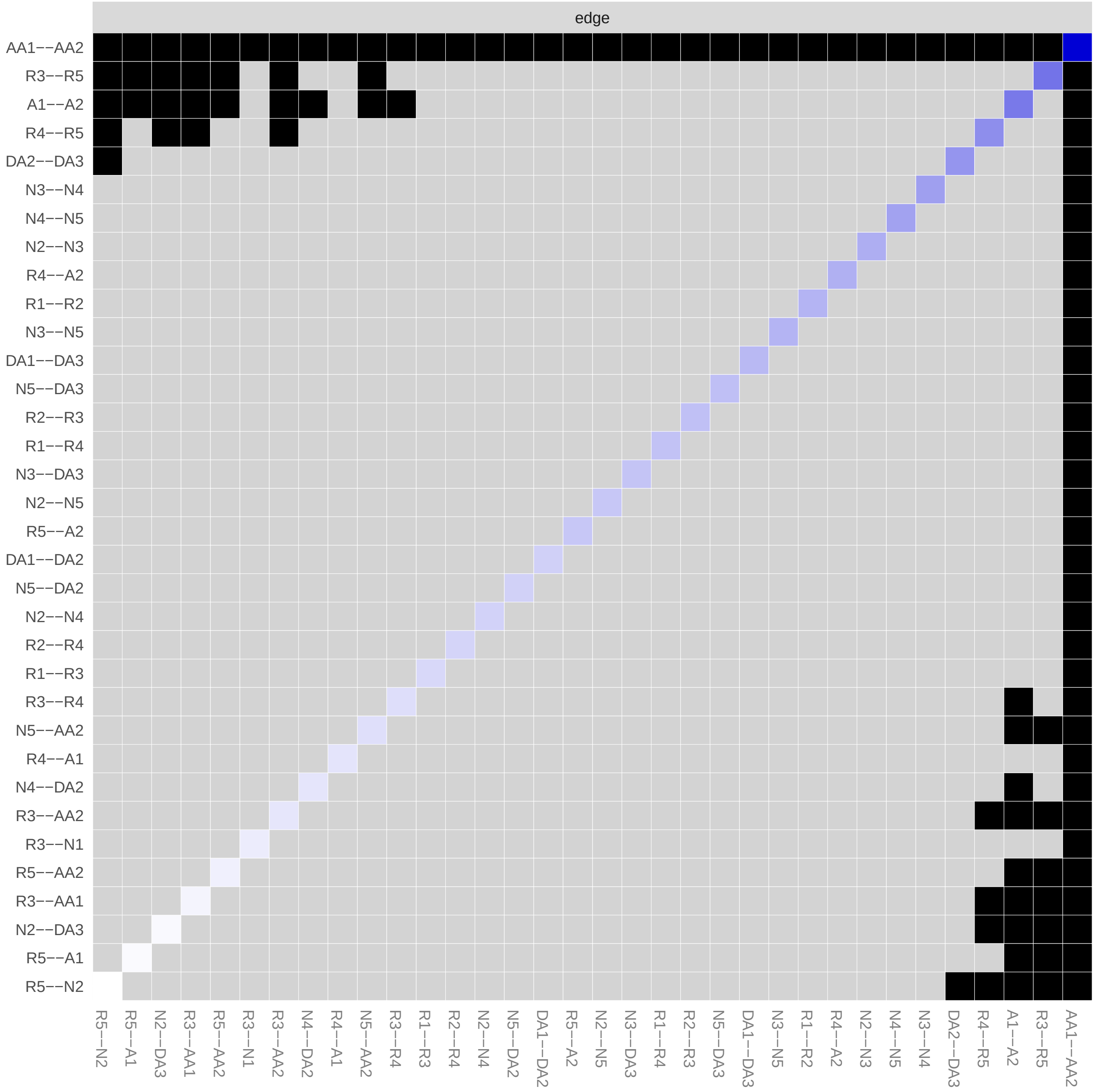

edge
AA1−−AA2
R3−−R5
A1−−A2
R4−−R5
DA2−−DA3
N3−−N4
N4−−N5
N2−−N3
R4−−A2
R1−−R2
N3−−N5
DA1−−DA3
N5−−DA3
R2−−R3
R1−−R4
N3−−DA3
N2−−N5
R5−−A2
DA1−−DA2
N5−−DA2
N2−−N4
R2−−R4
R1−−R3
R3−−R4
N5−−AA2
R4−−A1
N4−−DA2
R3−−AA2
R3−−N1
R5−−AA2
R3−−AA1
N2−−DA3
R5−−A1
R5−−N2

Figure S11.

Average correlations between centrality indices of networks of posttraumatic stress symptoms in women sampled with persons dropped and the original sample. Lines indicate the means and areas indicate the range from the 2.5th quantile to the 97.5th quantile.

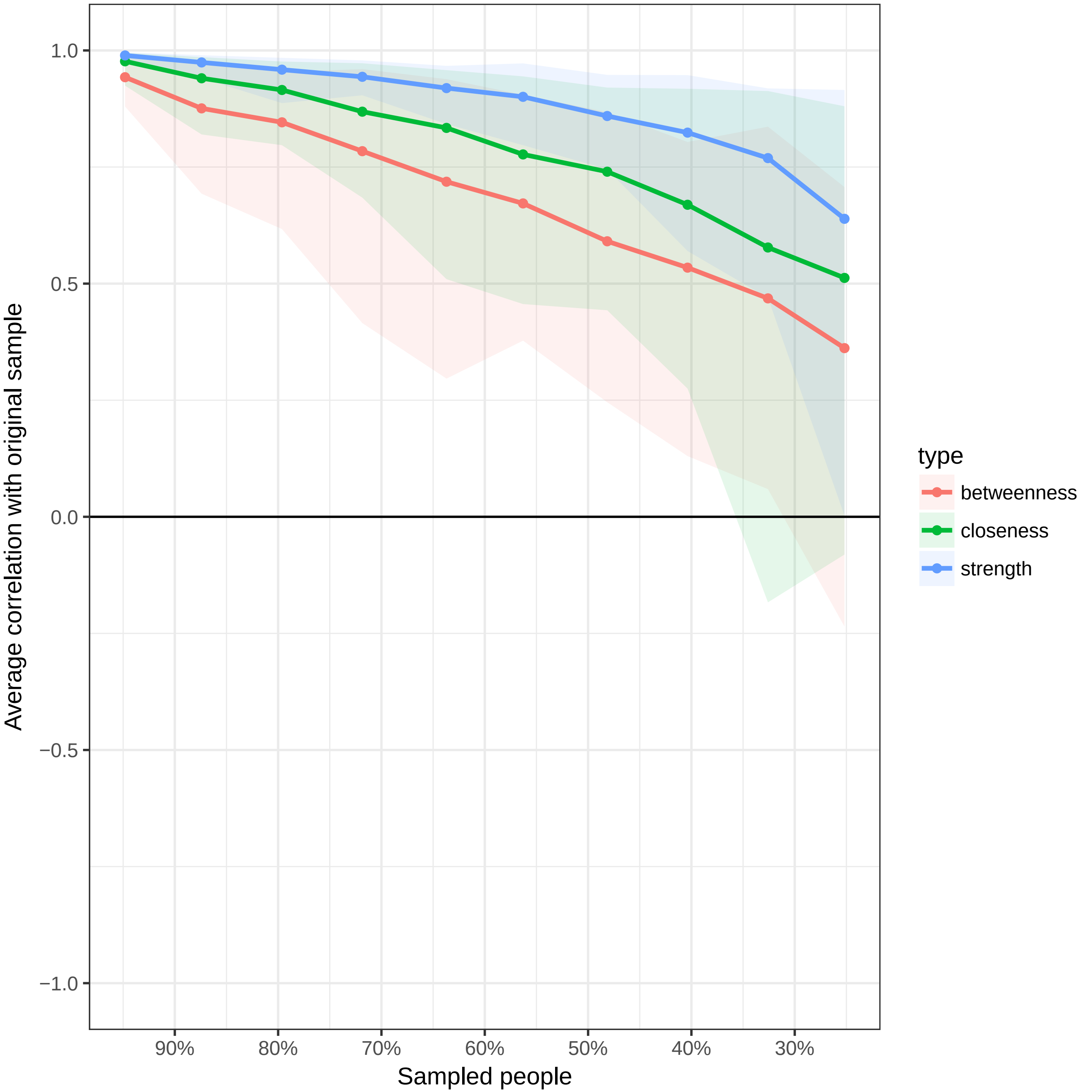

Average correlation with original sample
1.0
0.5
0.0
−0.5
−1.0
90%
80%
70%
60%
50%
40%
30%
Sampled people
type
betweenness
closeness
strength

Figure S12.

Average correlations between centrality indices of networks of posttraumatic stress symptoms in men sampled with persons dropped and the original sample. Lines indicate the means and areas indicate the range from the 2.5th quantile to the 97.5th quantile.

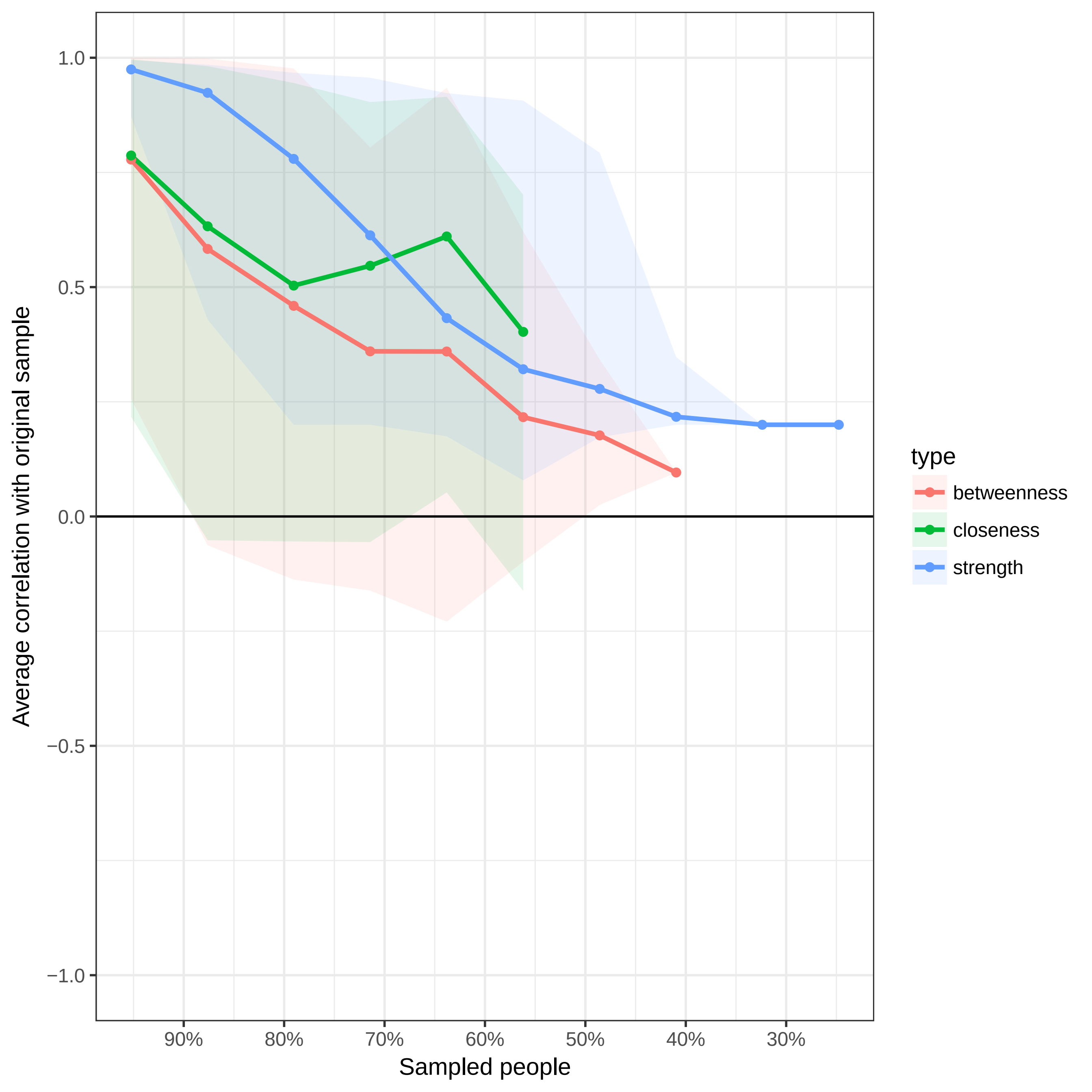

Average correlation with original sample
1.0
0.5
0.0
−0.5
−1.0
90%
80%
70%
60%
50%
40%
30%
Sampled people
type
betweenness
closeness
strength

Figure S13.

Bootstrapped difference tests (α = 0.05) between values of node strength for women. Gray boxes indicate strengths values that do not differ significantly from one-another and black boxes represent strengths values that do differ significantly from one-another.

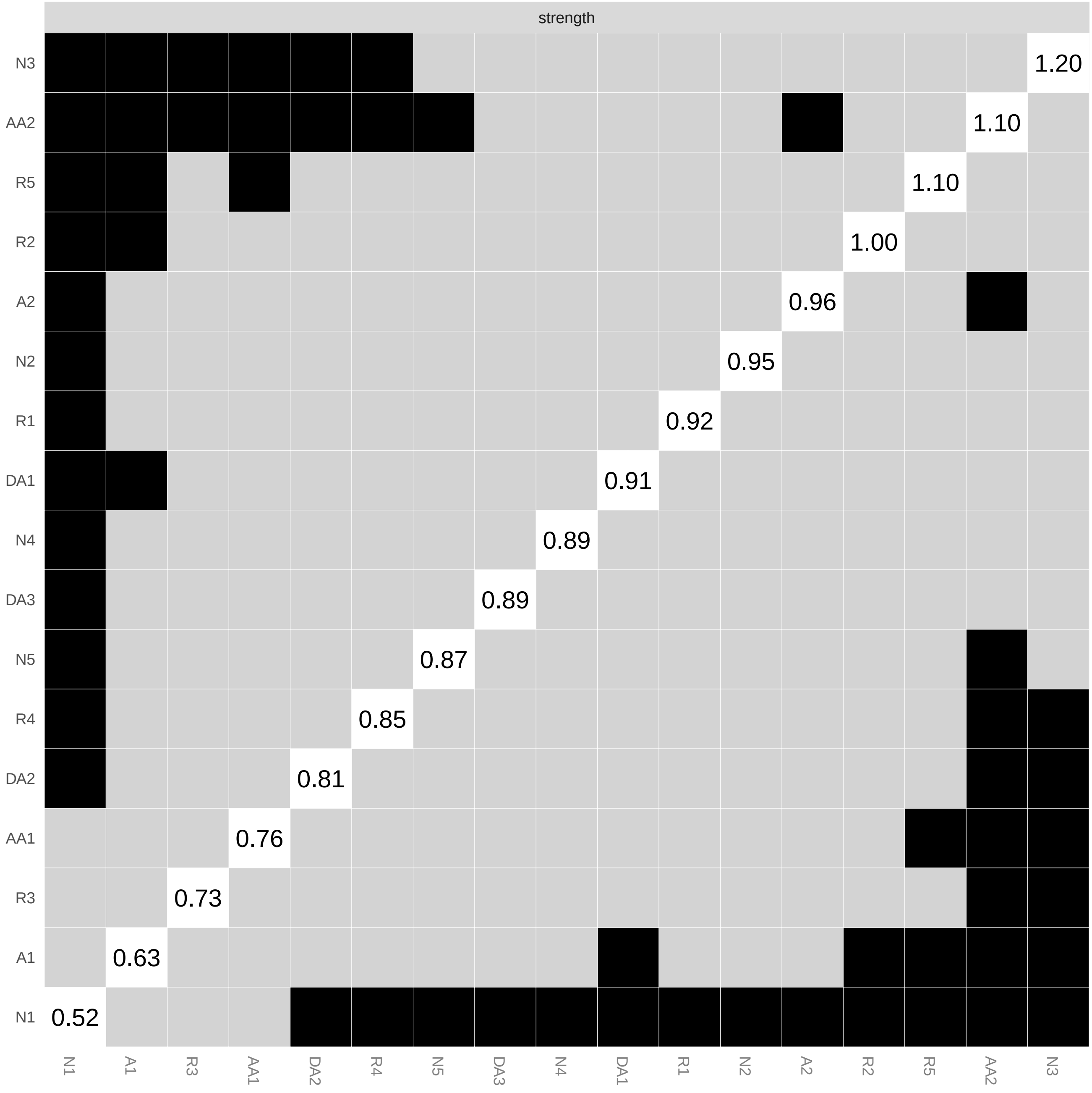
strength
N3
AA2
R5
R2
A2
N2
R1
DA1
N4
DA3
N5
R4
DA2
AA1
R3
A1
N1
1.20
1.10
1.10
1.00
0.96
0.95
0.92
0.91
0.89
0.89
0.87
0.85
0.81
0.76
0.73
0.63
0.52
N1
A1
R3
AA1
DA2
R4
N5
DA3
N4
DA1
R1
N2
A2
R2
R5
AA2
N3
