# Supplementary figures and images for: Gender Differences in Posttraumatic Stress Symptoms after a Terrorist Attack: A Network Approach
Source: Front Psychol. 2017 Dec 1;8:2091. doi: 10.3389/fpsyg.2017.02091 (PMC5717368; doi:10.3389/fpsyg.2017.02091)

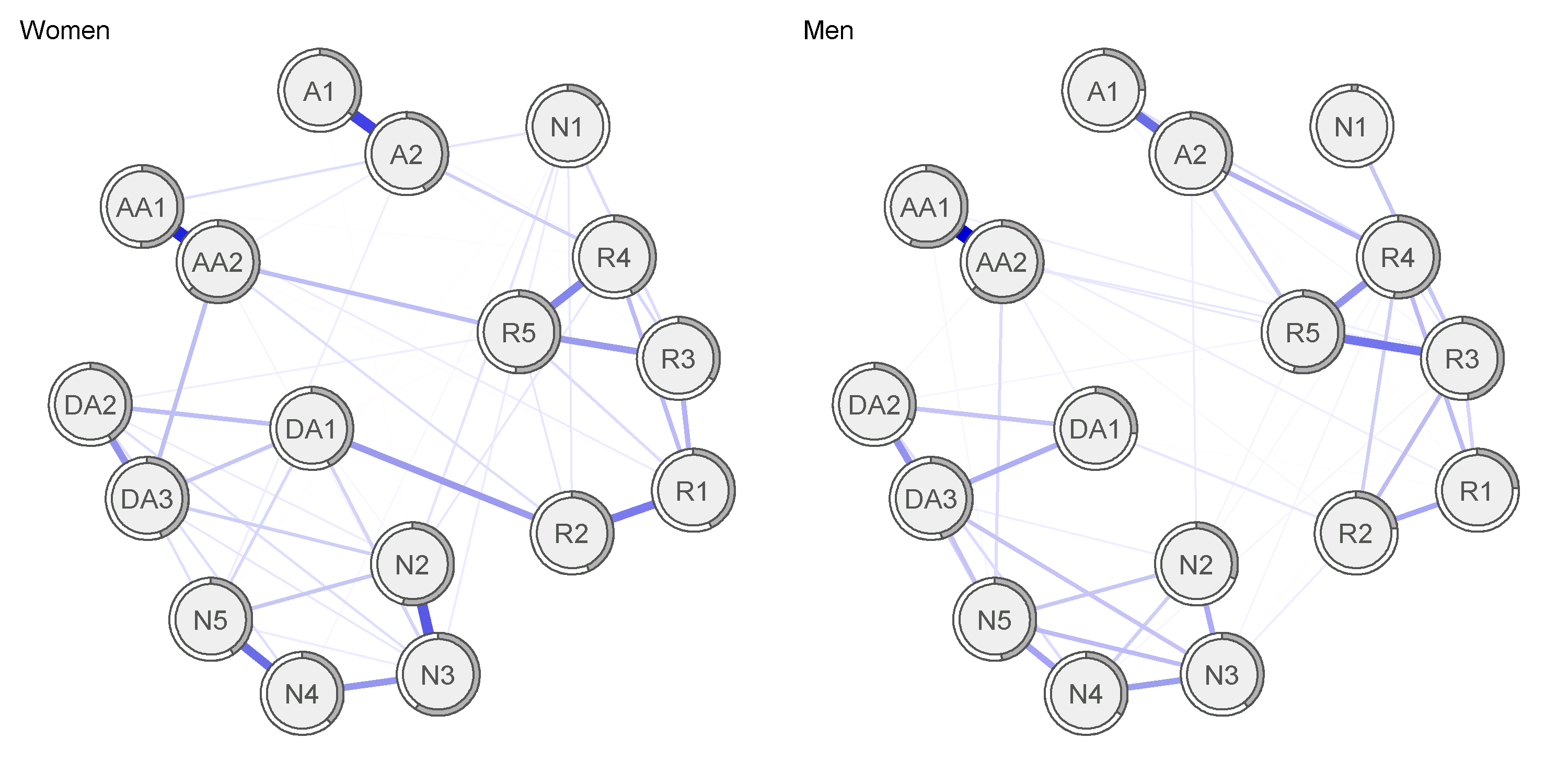

Supplement: Supplementary file 2 [file Presentation2.ZIP › Fig2.tiff]

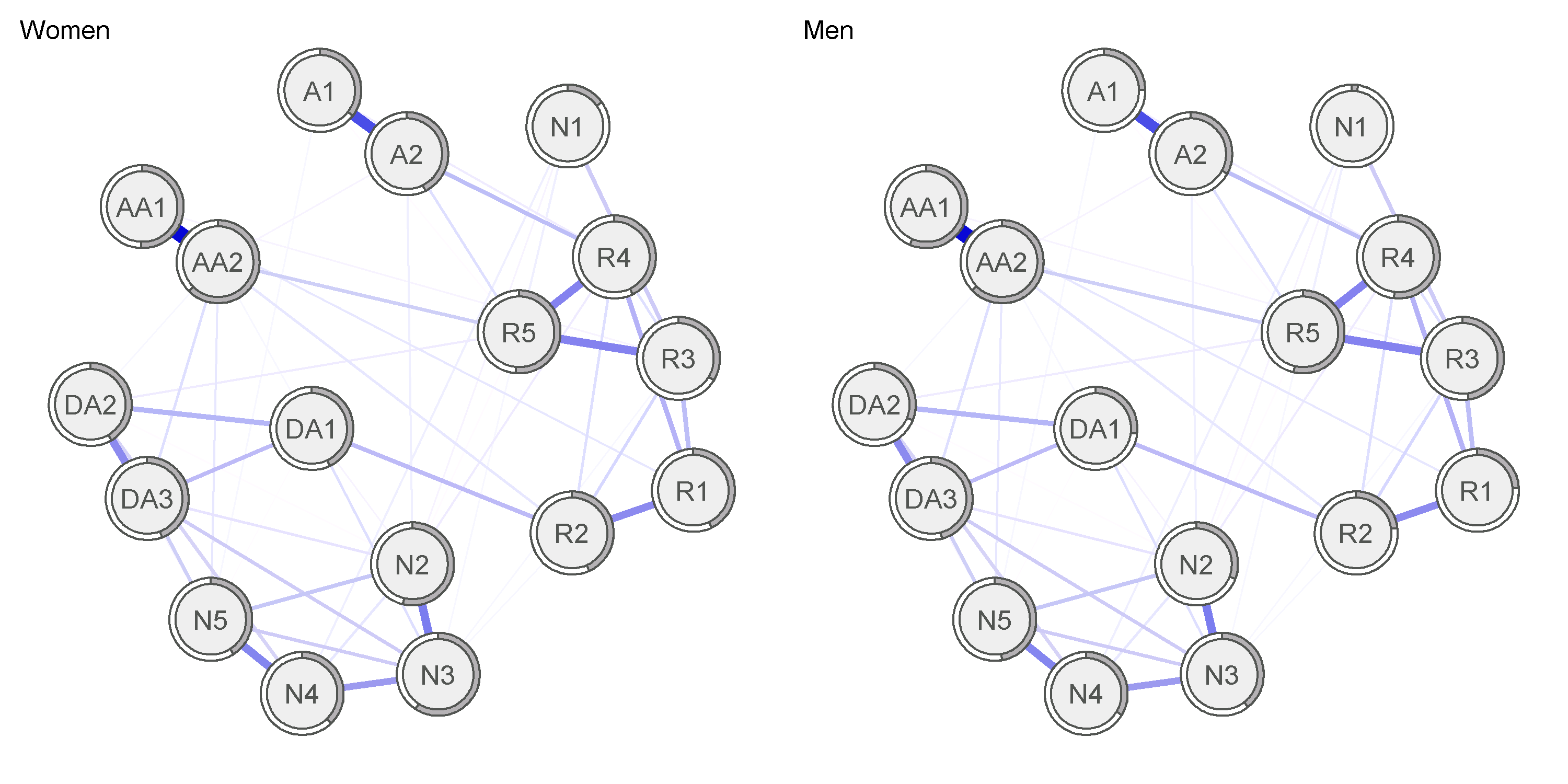

Supplement: Supplementary file 2 [file Presentation2.ZIP › Fig2_crossvalidation.tiff]

women, with ptsd

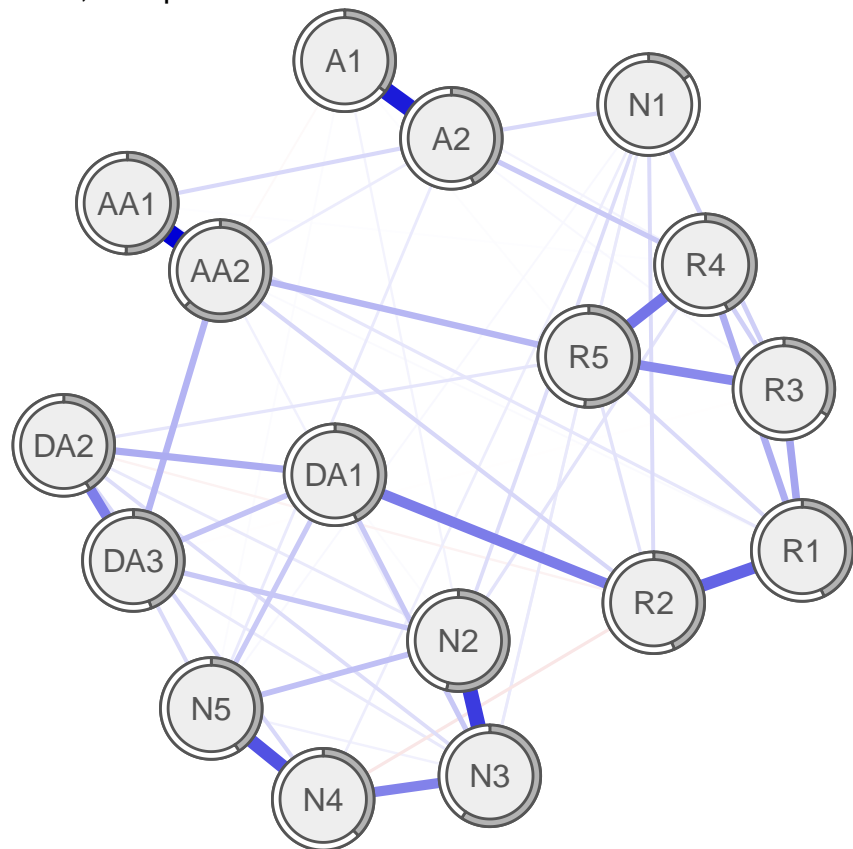

men, with ptsd

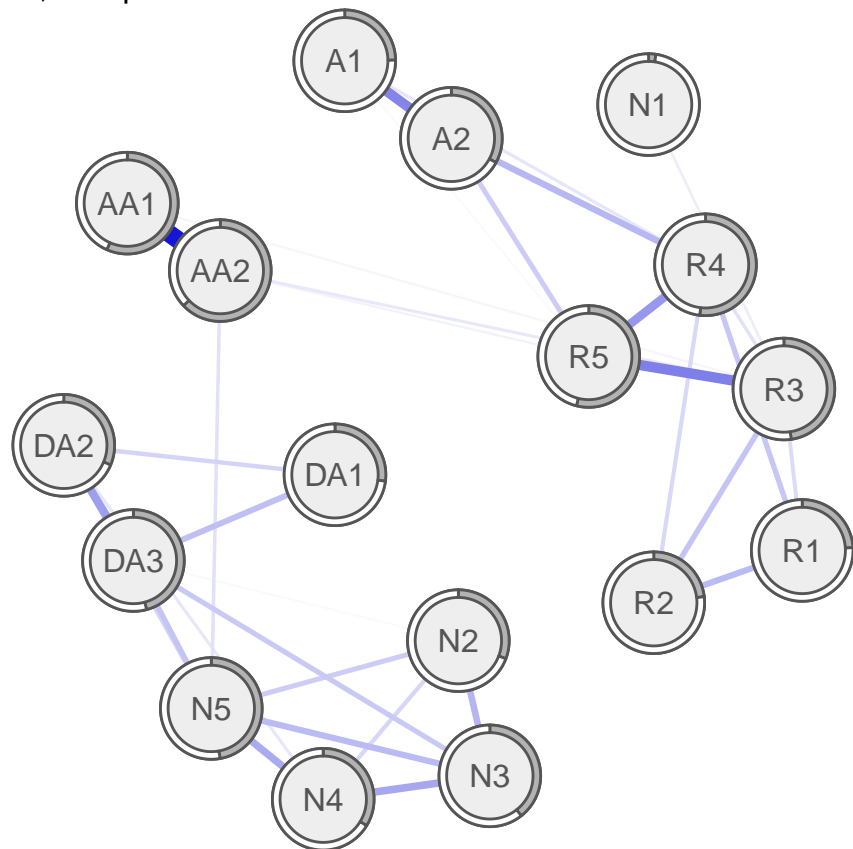

Supplement: Supplementary file 2 [file Presentation2.ZIP › individualnetworks.pdf]
